# Characterization of the SARS-CoV-2 antibody landscape in Norway in the late summer of 2022: high seroprevalence in all age groups with patterns of primary Omicron infection in children and hybrid immunity in adults

**DOI:** 10.1186/s12879-024-09670-w

**Published:** 2024-08-20

**Authors:** Gro Tunheim, Even Fossum, Anna Hayman Robertson, Gunnar Øyvind Isaksson Rø, Adity Chopra, John T. Vaage, Elisabeth Lea Vikse, Anne-Marte Bakken Kran, Per Magnus, Lill Trogstad, Siri Mjaaland, Olav Hungnes, Fridtjof Lund-Johansen

**Affiliations:** 1 Division of Infection Control, Norwegian Institute of Public Health (NIPH), Oslo, Norway; 2 Department of Immunology, Oslo University Hospital and University of Oslo, Oslo, Norway; 3 Center for Fertility and Health, Norwegian Institute of Public Health (NIPH), Oslo, Norway

**Keywords:** Seroprevalence, SARS-CoV-2, Antibodies, Children, Omicron, Wuhan, Infection, Vaccination, COVID-19, Nucleocapsid

## Abstract

**Background:**

According to Norwegian registries, 91% of individuals ≥ 16 years had received ≥ 1 dose of COVID-19 vaccine by mid-July 2022, whereas less than 2% of children < 12 years were vaccinated. Confirmed COVID-19 was reported for 27% of the population, but relaxation of testing lead to substantial underreporting. We have characterized the humoral immunity to SARS-CoV-2 in Norway in the late summer of 2022 by estimating the seroprevalence and identifying antibody profiles based on reactivity to Wuhan or Omicron-like viruses in a nationwide cross-sectional collection of residual sera, and validated our findings using cohort sera.

**Methods:**

1,914 anonymized convenience sera and 243 NorFlu-cohort sera previously collected from the Oslo-area with reported infection and vaccination status were analyzed for antibodies against spike, the receptor-binding domain (RBD) of the ancestral Wuhan strain and Omicron BA.2 RBD, and nucleocapsid (N). Samples were also tested for antibodies inhibiting RBD-ACE2 interaction. Neutralization assays were performed on subsets of residual sera against B.1, BA.2, XBB.1.5 and BQ.1.1.

**Results:**

The national seroprevalence estimate from vaccination and/or infection was 99.1% (95% CrI 97.0-100.0%) based on Wuhan (spike_W and RBD_W) and RBD_BA2 antibodies. Sera from children < 12 years had 2.2 times higher levels of antibodies against RBD_BA2 than RBD_W and their seroprevalence estimate showed a 14.4 percentage points increase when also including anti-RBD_BA2 antibodies compared to Wuhan-antibodies alone. 50.3% (95% CI 45.0-55.5%) of residual sera from children and 38.1% (95% CI 36.0-40.4%) of all residual sera were positive for anti-N-antibodies. By combining measurements of binding- and ACE2-RBD-interaction-inhibiting antibodies, reactivity profiles indicative of infection and vaccination history were identified and validated using cohort sera. Residual sera with a profile indicative of hybrid immunity were able to neutralize newer Omicron variants XBB.1.5 and BQ.1.1.

**Conclusions:**

By late summer of 2022, most of the Norwegian population had antibodies to SARS-CoV-2, and almost all children had been infected. Antibody profiles indicated that children mostly had experienced a primary Omicron infection, while hybrid immunity was common among adults. The finding that sera displaying hybrid immunity could neutralize newer Omicron variants indicates that Wuhan-like priming of the immune response did not have a harmful imprinting effect and that infections induce cross-reacting antibodies against future variants.

**Supplementary Information:**

The online version contains supplementary material available at 10.1186/s12879-024-09670-w.

## Background

When SARS-CoV-2 emerged in late 2019, the world’s population was immunologically naïve to the virus. Infection with SARS-CoV-2 induces antibodies against the viral spike glycoprotein and its receptor binding domain (RBD), as well as the internal nucleocapsid (N) protein [1]. By the end of 2020, vaccines against COVID-19 became available, initially containing the ancestral Wuhan spike protein as antigen, which induces antibodies against spike and RBD. Seroprevalence studies measuring antibodies against spike/RBD and N from SARS-CoV-2 can therefore be used to estimate the immune status in a population and/or the burden of infection. Whereas anti-spike and anti-RBD antibodies have been shown to be long-lasting, both after infection and vaccination [2, 3], anti-N antibodies have been shown to wane faster [4] and this could lead to underestimation of infections with increasing time since infection [5]. Moreover, studies have suggested that vaccinated individuals are less likely to develop anti-N antibodies after breakthrough infections [6, 7], probably as the virus is cleared more rapidly by anti-spike/RBD antibodies induced by the vaccine [7].

**Fig. 1:**
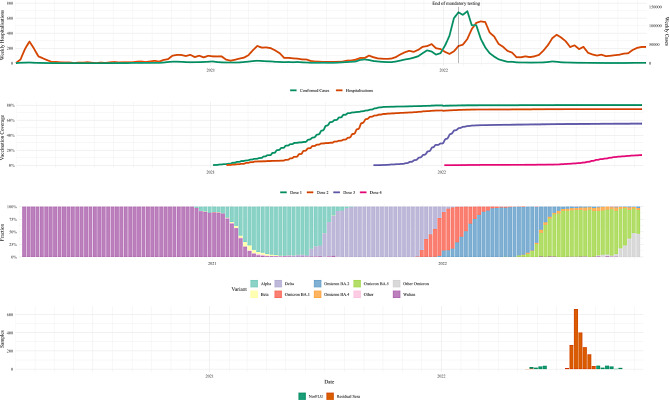
Overview of the COVID-19 pandemic and vaccination in Norway and study sampling periods. The upper panel shows the timeline of weekly numbers of laboratory confirmed SARS-CoV-2 cases and hospital admissions with COVID-19 as the main cause. The second panel from the top shows when the COVID-19 vaccination campaign started and vaccination coverage for doses 1–4. The second panel from the bottom shows the timeline for the virus variants, while the bottom panel shows the sampling period for residual sera and cohort sera in the present study. All timelines are aligned. The figure is based on public data from the Norwegian registries SYSVAK (vaccinations), MSIS (cases) and NoPaR (hospital admissions) [26] and on variant data collected by ECDC [27]

In Norway (population 5.4 million), there were two waves of SARS-CoV-2 infections in 2020: one in the spring and one in the autumn (Fig. 1) [8]. Non-pharmaceutical interventions, including a lock-down with closing of schools and social distancing in the spring of 2020, contributed to slowing the infection rate during the first year [9, 10]. In the spring of 2021, a third wave of infection occurred, largely due to the Alpha variant (B.1.1.7), which was subsequently replaced with the Delta variant (B.1.617.2) causing a fourth wave in the autumn. Like many other countries, Norway experienced its hitherto highest number of confirmed SARS-CoV-2 infections between the late autumn of 2021 and the spring of 2022 [11], predominantly after the introduction of the Omicron variant (B.1.1.529, abbreviated to BA. for its subvariants) in November 2021 [12]. Omicron is substantially different from the original Wuhan virus with several mutations in the spike and its RBD [13]. The Omicron sub-lineage BA.2 dominated between February and mid-June 2022 (Fig. 1) [11].

COVID-19 vaccines were first introduced in Norway in late December 2020 in a national vaccination program with vaccines given free of charge. Initially, prioritization was for health care workers, the elderly, and clinical risk groups, going downwards in age over time [9, 14]. Vaccinations are registered in the Norwegian Immunisation Registry (SYSVAK) (Fig. 1). The vaccines that were used were mostly mRNA vaccines from Moderna and Pfizer–BioNTech. Only ~ 2.4% of the Norwegian population received the AstraZeneca adenoviral vector vaccine as it was removed from the program in March 2021 due to severe side effects [15]. The Janssen vaccine was offered outside of the program [9] and inactivated vaccines were not used. Studies conducted prior to the introduction of the COVID-19 vaccines showed that the estimated seroprevalence in Norway increased from 0.8% (95% credible interval [CrI] 0.4-1.3%) in May 2020 to 3.2% (95% CrI 2.3%-4.2%) in January 2021 [8]. In August 2021, approximately 8 months into the national COVID-19 vaccine campaign, by which point all persons > 16 years had been offered two doses of the vaccine, the estimated seroprevalence was 62.6% (95% CrI 60.1%-65.2%) [14]. Vaccine uptake was in the same range; 58.4% [14]. In children under 12 years of age, the seroprevalence was 12.5% (95% CrI 9.3%-16.1%), mostly reflecting the burden of infection since this subpopulation was essentially unvaccinated. From September 2021, COVID-19 vaccination was extended to 12–15-year-olds (single dose). Finally, children aged 5–11 years were included in the national vaccination program from January 2022. However, vaccination was only recommended to children with specified risk conditions, and according to registry data, uptake was less than 2% in children < 12 years by mid-July 2022 [11, 16]. Overall, 78% of the population and 91% of individuals ≥ 16 years had received at least one dose of COVID-19 vaccine by mid-July 2022. Up until this point, only monovalent vaccines containing spike from the ancestral Wuhan strain were available through the national vaccination program.

From the start of the pandemic, all laboratory confirmed COVID-19 cases were registered in the Norwegian Surveillance System for Communicable Diseases (MSIS) (Fig. 1). At first, the testing capacity in Norway was limited and only symptomatic individuals fulfilling specific criteria were tested [8]. From the summer of 2020, PCR-testing was mandatory for symptomatic individuals and close contacts of COVID-19 cases. However, in January 2022, changes in the national testing strategy led to increased use of self-administered rapid antigen tests that were not registered in MSIS, and eventually testing was no longer mandatory [11]. Even prior to the changes in the testing strategy, we observed that a large proportion of infections had gone unregistered [14]. Since these relaxations in testing coincided with the large Omicron wave, we anticipate that the number of registered cases substantially underrepresent the actual number of infections. In mid-July 2022, 27% of the population was registered as having been infected in MSIS [11].

Here, we have characterized the SARS-CoV-2 antibody landscape in Norway in the late summer of 2022 after widespread circulation of the first Omicron strains. Firstly, we have estimated the SARS-CoV-2 seroprevalence in Norway by measuring antibodies against spike and RBD from the ancestral Wuhan strain, and RBD from the BA.2 Omicron strain, using a nationwide, cross-sectional collection of anonymized residual sera sampled mainly in August 2022 (Fig. 1). Secondly, we present a more in-depth analysis of the humoral immunity of the Norwegian population by also measuring anti-N antibodies and antibodies inhibiting the interaction between the receptor angiotensin-converting enzyme 2 (ACE2) and RBD as a proxy of neutralizing antibodies, subsequently validated by live virus neutralization assays. Based on the analyses of the antibody responses in the residual sera, we identified antibody reactivity profiles likely to reflect infection and vaccination history. Sera collected during a similar time period from participants in a prospective cohort study (NorFlu) (Fig. 1) with information on vaccination and infection status were used to validate these antibody profiles.

## Methods

### Study samples

#### Residual sera

Between week 20 and 36 (16 May-11 September ) 2022, 1,914 anonymized residual sera were collected using convenience sampling by 17 laboratories across Norway, as previously described (Fig. 1) [14, 17]. The laboratories were asked to participate with approximately 120 sera collected between weeks 31–35 and obtain a prespecified number of samples in the age groups 0–4, 5–14, 15–24, 25–59 and ≥ 60 years, excluding sera from individuals with known or suspected hepatitis B or C or HIV. The sera were therefore sampled based on the geographical location, age categories and available volume of the residual sera (at least 0.3 ml for children and at least 1 ml for adults). Data on birth year, sex, and county of residence was recorded.

#### Samples from cohort participants with reported infection and/or vaccination history

Samples already collected through an existing cohort, the Norwegian Influenza Pregnancy Cohort (NorFlu), were utilized retrospectively for validation of the results from the residual sera. NorFlu is a prospective population-based longitudinal cohort initiated during the A/H1N1 influenza pandemic in 2009/2010 [18] whereby pregnant mothers with their offspring (born in 2010) were included. In 2020, a COVID-19 focusing sub-study was initiated, and all NorFlu study participants were asked to answer biweekly questionnaires on infection and vaccination [19]. Between 23 May-13 October 2022, a subset of the mothers and their families, resident in the Oslo area, were invited to donate blood samples at Oslo University Hospital in order to study humoral and cellular immunity after COVID-19 infection and vaccination. In total, 218 mothers, their NorFlu child (aged 11–12 years), together with the children’s fathers, and siblings aged 8–15 years were invited. All participants were asked to complete a questionnaire on infection history at the time of sampling. The participants (*n* = 243) were further divided into two age categories for analysis: ≤12 years (*n* = 90) and > 12 years (*n* = 153), from here on referred to as the “cohort children” and “cohort adults”, respectively. In contrast to the age group “<12 years” for residual sera, NorFlu participants aged 12 years were included as children.

#### Registry data on vaccination and infection

In Norway, all vaccinations are registered in the Norwegian Immunisation Registry (SYSVAK). All COVID-19 vaccines used in Norway until late autumn of 2022 were monovalent containing the ancestral Wuhan strain and mRNA vaccines were almost exclusively used. Since the start of the pandemic, all laboratory-confirmed COVID-19 cases have been registered in the Norwegian Surveillance System for Communicable Diseases (MSIS). However, due to increased use of rapid antigen tests performed at home from January 2022 and later cancellation of mandatory testing, the number of confirmed cases reported to MSIS is not representative of the total number of cases. Aggregated COVID-19 data from SYSVAK and MSIS are made publicly available in weekly reports [11].

#### Definition of exposure groups for cohort participants

For NorFlu cohort participants, records of dates and doses of COVID-19 vaccination were obtained from SYSVAK. Information on confirmed SARS-CoV-2 infection and dates of infection were obtained from MSIS (laboratory-confirmed) and from self-reported questionnaires (confirmed by rapid antigen assay through self-testing). SYSVAK data, MSIS data, questionnaire data and antibody analysis results were linked using the participants national identity number, a unique identification number issued to all Norwegian residents.

The infection history is based on participants self-selecting to get tested in accordance with the changing government policy on testing. Although testing was no longer mandatory after January 2022, almost 50% of the primary infections, and the majority of reinfections, were reported after this time point. Participants for whom information on infection history during the Omicron wave was missing (*n* = 14; 11 fathers and 3 siblings) were excluded in the antibody-profiling analysis of participants with reported infection/vaccination history, as were participants who were uninfected and unvaccinated (5 children), giving a total of *n* = 224.

Dates of infection were used to determine the number of infections per individual, with a minimum of 30 days between each first positive test. Time periods for circulation of predominating strains were obtained from the national virological surveillance system (NIPH) (Fig. 1). Wuhan, Alpha and Delta strains predominantly circulated from 2020 until the end of 2021 and were grouped as “Wuhan-like” strains, whereas the variants BA.1, BA.2 and BA.5 which dominated from January 2022 were grouped as “Omicron” strains. Each cohort participant’s infection was assigned a virus type according to their date of infection, and each individual further assigned an exposure category based on their infection and vaccination history, as follows: (1) infected with Wuhan-like strain, unvaccinated (*n* = 13), (2) infected with Wuhan-like strain and later with Omicron, unvaccinated (*n* = 12), (3) infected with Omicron, unvaccinated (*n* = 56), (4) Omicron infection and later vaccinated (*n* = 1), (5) vaccinated, non-infected (*n* = 30), (6) vaccinated and infected with Wuhan-like strain (*n* = 19; of which 2 were infected before and 17 after vaccination), and (7) vaccinated and subsequently infected with Omicron (*n* = 93, of which 4 were infected with a Wuhan-like strain before vaccination). Finally, for participants with a single infection, sampling and infection dates were used to determine the time since infection in days.

### Measurement of binding antibodies

Serum IgG against the full-length spike protein and RBD from ancestral Wuhan-Hu-1 (Spike_W and RBD_W, respectively), Omicron BA.2 RBD (RBD_BA2) and N was measured using a multiplex bead-based flow cytometry assay [20]. Antibody levels were measured as median fluorescent intensity (MFI) of beads coupled with viral antigens relative to beads with no antigen (relative MFI; rMFI). Samples above the cut-off rMFI values for each antigen were considered seropositive for the corresponding antigen, as previously described [14].

### ACE2-RBD interaction assay as proxy for neutralization

The inhibitory effect of serum on binding of ACE2 to RBD_W and RBD_BA2 was measured as previously described [20]. Sera were incubated with beads coupled with RBD_W or RBD_BA2. After centrifugation and removal of serum, digoxigenin-labelled recombinant ACE2 was added to the beads. After incubation and washing, monoclonal anti-Digoxin conjugated to R-Phycoerythrin was added. If the serum contains antibodies binding to RBD epitopes involved in the ACE2-RBD interaction, the binding of ACE2 will be inhibited [20]. Percent inhibition was calculated using beads without serum as reference for “no inhibition”.

### Neutralization assay

Neutralization assays were performed using isolates of SARS-CoV-2 variants B.1 (available in GISAID EpiCoV with accession numbers EPI_ISL_449791), BA.2 (EPI_ISL_16100571), BQ.1.1 (EPI_ISL_15349765) and XBB.1.5 (EPI_ISL_16969674) as previously described [21]. Three subsets of 20 residual sera each were manually selected to best represent the ability to inhibit RBD-ACE2 interaction of RBD_W (RBD_BA2-ACE2/RBD_W-ACE2 ratio > 10, median = 18.6), RBD_BA2 (RBD_BA2-ACE2/RBD_W-ACE2 ratio < 0.3, median = 0.04), or both (RBD_BA2-ACE2/RBD_W-ACE2 ratio 1–10, median = 2.8). Selected sera were heat-inactivated for 30 min at 56 °C and serially diluted twofold. In a BSL-3 facility, a viral dose of 100xTCID_50_ was added to each well of diluted sera and to a virus control not containing serum. Cell controls only containing virus diluent were also included. The virus-serum mix was incubated at 37 °C for 1 h and then added to Vero E6 cells (12,000 cells/well). After 96 h of incubation at 37 °C the cells were fixed.

An ELISA detecting the N protein of SARS-CoV-2 was performed on the fixed cell layer in a BSL2 facility as described in [21]. The 50% virus neutralization titer was calculated from the measured OD values using GraphPad Prism version 9 (GraphPad Software, Boston, Massachusetts USA). For statistical analysis, serum samples that did not yield any neutralization at dilution 1:10 were assigned a titer of 5.

### Statistical analyses

As previously described, seroprevalence was estimated for Norway and by age groups (also for cohort sera), sex, and county of residence using Bayesian statistics [14, 22]. We estimated seroprevalence using two methods. In method 1, we only consider seropositivity against the Wuhan variant (having antibodies against both spike_W and RBD_W). This is the same method as previously published [14], where we could use an estimated sensitivity and specificity based on known negatives and known positives. For method 2, we consider seropositivity against either the Wuhan (both spike_W and RBD_W antibodies) or the Omicron BA.2 (having RBD_BA2 antibodies) variants where we do not have good measurements for the sensitivity or specificity of the test against BA.2. To estimate the seroprevalence in this case we extend the model for the one-variant case by denoting the probability of being positive on either one or both of the tests as $\:p$ which is related to $\:{p}_{1}$, the probability of being positive against the Wuhan strain, $\:{p}_{2}$, the probability of being positive against the BA.2 strain and $\:{p}_{12}$, the probability of being positive against both strains.

$$\:p={p}_{1}+{p}_{2}+{p}_{12}.$$

To estimate $\:p$ we use the following model:

$$\:{Y}_{1}\:\sim\:binom(N,{p}_{1}{s}_{1}\:+\:\left(1-{p}_{1}\right)*\left(1-{c}_{1}\right))$$

$$\:{Y}_{2}\sim\:binom\left(N,{p}_{2}{s}_{2}+\left(1-{p}_{2}\right)\left(1-{c}_{2}\right)\right)$$

$$\:{Y}_{12}\sim\:binom\left(N,{p}_{12}{s}_{12}+\left(1-{p}_{12}\right)\left(1-{c}_{12}\right)\right)$$

$$\:{p}_{12}=max\left(0,{p}_{2}+{p}_{1}-1\right)+{p}_{a}\left(min\left({p}_{1},{p}_{2}\right)+max\left(0,{p}_{2}+{p}_{1}+1\right)\right)$$

$$\:{s}_{2}\sim\:normal\left({s}_{1},{\upsigma\:}\right)$$

$$\:{s}_{12}\sim\:normal\left({s}_{1},{\upsigma\:}\right)$$

$$\:{c}_{2}\sim\:normal\left({c}_{1},{\upsigma\:}\right)$$

$$\:{c}_{12}\sim\:normal\left({c}_{1},{\upsigma\:}\right)$$

Where $$\:{Y}_{i}$$ are the number of people testing positive for the different combinations of tests $$\:i=1,\:2,\:12$$. $$\:{s}_{i}$$ and $$\:{c}_{i}$$ are the unknown sensitivity and specificity with $$\:{s}_{1}$$ and $$\:{c}_{1}$$ defined and modelled as previously published [2]. $$\:{p}_{1}$$$$\:{p}_{2},$$ and $$\:{p}_{a}$$ are auxiliary estimated parameters between 0 and 1. We estimate $$\:{p}_{a}$$ instead of $$\:{p}_{12}{}_{}$$ directly since $$\:{p}_{12}$$ cannot be large than either $$\:{p}_{1}\:$$or $$\:{p}_{2}\:$$and has to be larger than $$\:{p}_{2}+{p}_{1}-1$$ to ensure that$$\:\:p$$ remains below 1. The $$\:{\upsigma\:}$$ parameter controls how close to the sensitivity and specificity for the BA.2 and the combined tests are to the characteristics from method 1; we choose $$\:{\upsigma\:}=\:0.05$$. The model is implemented in the STAN [23] probabilistic programming language using the Rstan interface [24]. The models are available at GitHub [25].

To estimate the overall seroprevalence we performed a multilevel regression and post stratification analysis as previously described [14] for method 1 and similarly for method 2 by using the model described above. Seroprevalence was not estimated if a subgroup had less than 30 samples.

95%confidence intervals (CI) for proportions were calculated using the exact method as previously described [14]. Reported correlations are Spearman correlations with confidence intervals estimated by the bootstrap method all calculated using Stata/SE 17.0 for Windows (StataCorp, College Station, Texas, USA). Significant differences in neuralization titers between subsets of residual sera were calculated using Kruskal-Wallis test with Dunn`s multiple comparison test (GraphPad Prism version 9).

For the cohort sera, anti-N antibody levels amongst confirmed COVID-19 cases (limited to one infection episode) were plotted against the number of days between infection and date of sampling for vaccinated and unvaccinated individuals respectively to explore antibody waning over time in these two groups. Linear regression was used to plot the best line of fit with 95 CI, and to estimate the association between anti-N antibody levels over time and vaccination status (in Stata version 18, Stata Corp, Texas, USA).

### Ethics

This study was approved by the Regional Committee for Medical and Health Research Ethics in Southeastern Norway (REC) (reference number 157792 for use of the residual sera and reference number 18403 for the NorFlu cohort). For the residual sera, which were irreversibly anonymized before they were receive at the Norwegian Institute of Public Health, written informed consent is not required by Norwegian law and this has been specifically approved by REC. Written informed consent was obtained from all NorFlu cohort participants. For minors (NorFlu) written informed consent was obtained from both parents. The study was conducted in accordance with the Declaration of Helsinki.

## Results

### Seroprevalence estimates for the Norwegian population based on residual sera

In the summer of 2022, 1,914 residual sera were collected from laboratories representing all 11 Norwegian counties. Most of the sera (90.7%) were collected between weeks 30 to 34 (25 July to 28 August) (Fig. 1) (Supplementary Table 1). For 123 samples (6.4%), the collection week was missing. The range of samples submitted per laboratory was 77–120. The donors of the residual sera were aged 0 to 96 years with a median age of 29 years and 54.5% of the samples were from females.

Most of the serum samples (92.8% (95% CI 91.5%-93.9%)) were positive for antibodies against the Wuhan variant of SARS-CoV-2 (both spike_W and RBD_W antibodies) (Table 1). However, children < 12 years were less likely to be seropositive than older individuals (Supplementary Table 2). Based on samples positive for antibodies against the Wuhan variant, the national seroprevalence estimate was 95.5% (95% CrI 93.6%-97.7%) (method 1, Table 1). The seroprevalence estimate for children < 12 years was lower; 83.3% (95% CrI 79.0%-87.5%) (Supplementary Table 2).

**Table 1:** Seroprevalence estimates and vaccine coverage, overall and by age groups and sex, late summer of 2022

	Number of samples tested (%)	Positive samples, method 1^a^ (Wuhan antibodies)	Positive samples, % (95% CI), method 1	Seroprevalence estimate, % (95% credible interval), method 1	Positive samples, method 2^b^ (Wuhan & BA.2 antibodies)	Positive samples, % (95% CI), method 2	Seroprevalence estimate, % (95% credible interval), method 2	Vaccinated with at least one dose of COVID-19 vaccine, (%). Data from reference no. 11
National (all ages)	1,914 (100)	1,776	92.8 (91.5–93.9)	95.5 (93.6–97.7)	1,852	96.8 (95.9–97.5)	**99.1 (97.0–100.0)**	78
**Age groups (year)**								
0–4	180 (9.4)	143	79.4 (72.8–85.1)	81.4 (75.2–87.1)	163	90.6 (85.3–94.4)	96.8 (89.8–100.0)	0^c^
5–11	186 (9.7)	154	82.8 (76.6–87.9)	84.9 (78.5–90.5)	176	94.6 (90.3–97.4)	98.2 (93.7–100.0)	2
12–17	229 (12.0)	212	92.6 (88.4–95.6)	95.0 (90.8–98.9)	222	96.9 (93.8–98.8)	99.0 (96.6–100.0)	55/84^d^
18–44	701 (36.6)	674	96.1 (94.4–97.4)	98.5 (96.6–99.9)	688	98.1 (96.8–99.0)	99.6 (98.5–100.0)	88
45–64	330 (17.2)	316	95.8 (93.0–97.7)	98.0 (95.4–99.9)	323	97.9 (95.7–99.1)	99.5 (98.1–100.0)	93
≥ 65	288 (15.0)	277	96.2 (93.3–98.1)	98.2 (95.6–99.9)	280	97.2 (94.6–98.8)	99.5 (98.0–100.0)	96
**Sex**								
Female	1,044 (54.5)	969	92.8 (91.1–94.3)	95.5 (93.2–98.0)	1,008	96.6 (95.3–97.6)	99.0 (96.8–100.0)	81^e^
Male	868 (45.4)	806	92.9 (90.9–94.5)	95.5 (93.1–98.2)	842	97.0 (95.6–98.0)	99.1 (97.0–100.0)	79^e^
Missing	2 (0.1)	1	50.0 (1.3–98.7)	n.a.	2	100.0 (15.8–100.0)	n.a.	50.0 (1.3–98.7)

n.a.: not applicable. Seroprevalence was not estimated if a subgroup had less than 30 samples

^a^Method 1: seropositivity was based on having antibodies against both spike and receptor binding domain (RBD) from the Wuhan variant of SARS-CoV-2. ^b^Method 2: seropositivity was based on either having antibodies against both RBD and spike from the Wuhan variant of SARS-CoV-2 (as in Method 1) or having antibodies against Omicron BA.2 RBD. ^c^The COVID-19 vaccines in use until the autumn of 2022 were not approved for children < 5 years. ^d^55% for 12-15-year-olds, 84% for 16-17-year-olds. ^e^Data from the end of week 28 2022, provided by Kristian Lie and Trude M. Lyngstad; the total number of women and men are from January 1, 2022

**Fig. 2:**
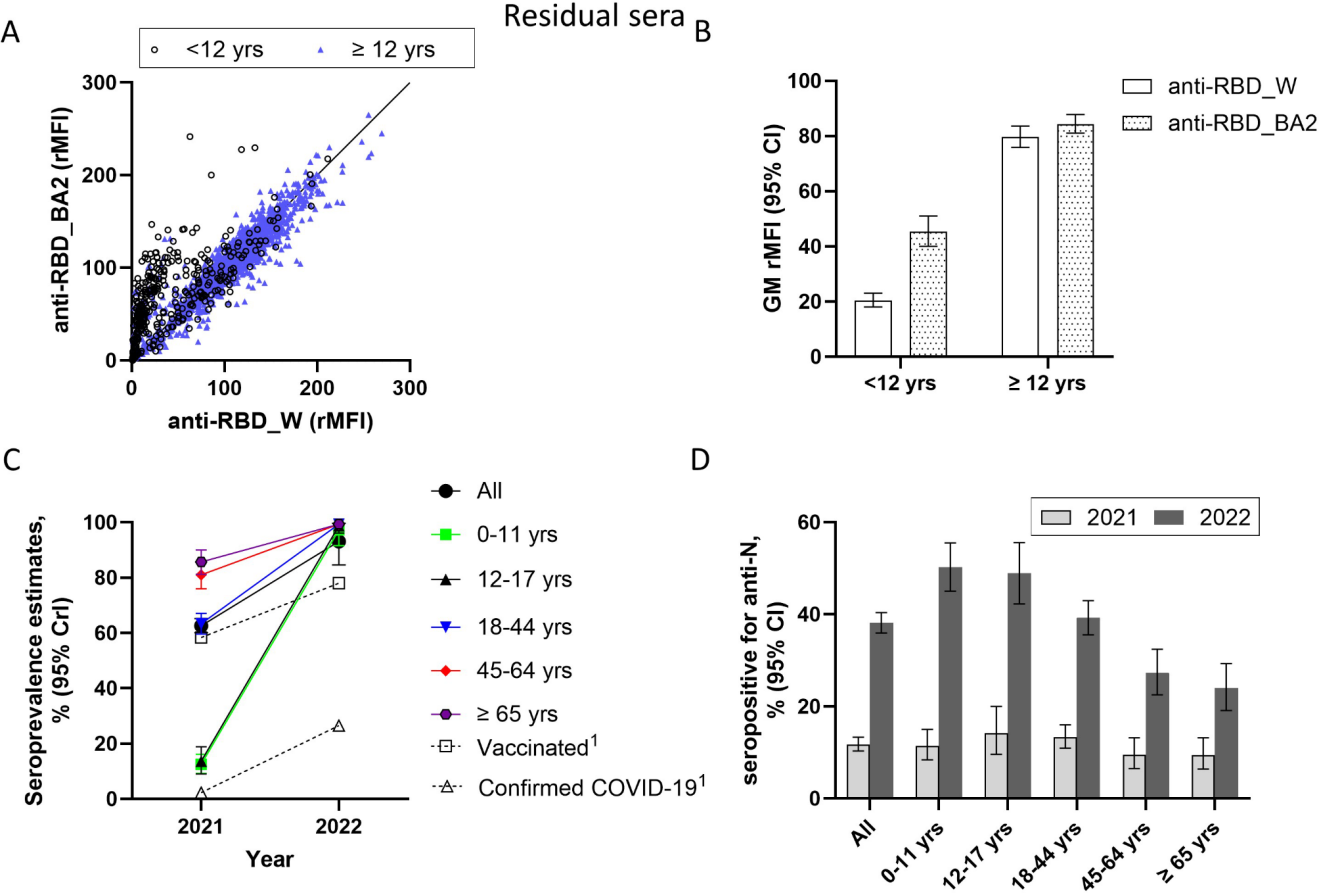
Antibodies against SARS-CoV-2 in residual sera and corresponding national seroprevalence estimates. **A**) and **B**) Antibodies against RBD from ancestral Wuhan (RBD_W) or Omicron BA.2 (RBD_BA2) variants. Antibody levels are given as relative median fluorescent intensity (rMFI). In A) each circle represents one individual. **B**) Geometric means (GM) of rMFI with 95% CI are shown. **C**) Changes in seroprevalence estimates with 95% CrI from August 2021 [2] to the late summer of 2022, overall and according to age groups. Changes in COVID-19 vaccination and infection is also shown [2, 4]. D) Proportion of samples positive for antibodies against nucleocapsid (N) with 95% CI in August 2021 [2] and the late summer of 2022, overall and according to age groups

Due to the large wave of Omicron infections in the spring of 2022 (Fig. 1) [11], we also tested the residual sera for antibodies against RBD_BA2. Interestingly, we observed that 76 of the residual sera (4.0%) had antibodies against RBD_BA2 but were not seropositive for antibodies (spike_W and RBD_W) against the Wuhan variant. This antibody pattern was more common in samples from children < 12 years than for samples from individuals ≥ 12 years (11.5% versus 2.2%, respectively). The Spearman correlation coefficient for these two RBD antibody types was 0.782 (95% CI 0.737–0.827) for the children’s samples, while in samples from older individuals, the correlation coefficient was higher (0.939 (95% CI 0.929–0.948). Moreover, the geometric mean (GM) of the rMFI was 2.2 times higher for antibodies against RBD_BA2 than RBD_W for children < 12 years (Fig. 2A & B). For individuals ≥ 12 years, the antibody levels against RBD_BA2 and RBD_W were more similar. Thus, if seropositivity was based on having antibodies against the Wuhan variant (RBD_W and spike_W), as previously published [2], the seroprevalence against SARS-CoV-2 in the Norwegian population would be underestimated, particularly for children. We therefore updated the seroprevalence estimates to also include samples with antibodies against RBD_BA2 only (i.e., using method 2). The updated seroprevalence estimate for Norway in the late summer of 2022 was 99.1% (95% CrI 97.0%-100.0%) (Table 1), i.e., resulting only in a small increase. However, the effect of including these antibodies in the seroprevalence estimates was higher in the youngest age groups (Table 1). For individuals < 12 and ≥ 12 years, the updated seroprevalence estimates were 97.7% (95% CrI 92.6%-100.0%) and 99.5% (95% CrI 98.2%-100.0%), respectively (Supplementary Table 2), thus, the estimates for these age groups were more similar using the updated method.

The SARS-CoV-2 seroprevalence estimates were considerably higher in the late summer of 2022 than in August 2021 (Fig. 2C) [14]. All age groups displayed an increase in their seroprevalence estimate since August 2021 with the largest differences in the younger age groups. In August 2021, the estimated seroprevalence was 12.5% (95% CrI 9.3–16.1%) for children < 12 years. The updated estimate from 2022 indicated that 85% of children in this age group have changed from being seronegative to being seropositive over the course of one year.

The seroprevalence estimates from infection and/or vaccination were similar for females and males (Table 1). All updated county seroprevalence estimates were ≥ 98% [28]and there were no differences in seroprevalence estimates between the 11 Norwegian counties (Supplementary Table 3). Correspondingly, the vaccination coverage of all counties for the first dose of COVID-19 vaccine was ≥ 90% for individuals ≥ 16 years by the 22 May 2022 [28]. Seroprevalence estimates were similar between sampling weeks as well as for samples with missing sampling week (Supplementary Table 1).

### Anti-N antibodies in residual sera

SARS-CoV-2 infection may induce antibodies against the N protein. Overall, 38.1% (95% CI 36.0%-40.4%) of the residual sera had antibodies against N indicative of previous SARS-CoV-2 infection, compared with 11.7% (95% CI 10.3%-13.3%) in August 2021 [14] (Fig. 2D). The proportion of anti-N positive samples decreased by age; 50.3% (95% CI 45.0%-55.5%) of samples from children < 12 years were positive, while 35.3% (95% CI 32.9%-37.7%) of samples from older individuals were positive. There were greater differences between the age groups in 2022 than in 2021 (Fig. 2D). The correlation coefficients between anti-N levels and anti-RBD_W/RBD_BA2 levels were higher in sera from individuals < 12 years (0.625 (95% CI 0.559–0.690)/0.673 (95% CI 0.612–0.734)) than from older individuals (0.215 (95% CI 0.166–0.265)/0.266 (95% 0.218–0.315)). For children aged < 12 years (less than 2% vaccination coverage), there was a 42% absolute difference between the seroprevalence estimate according to method 2 and the proportion of samples positive for N.

### Inhibition of ACE2-RBD interaction by residual sera

**Fig. 3:**
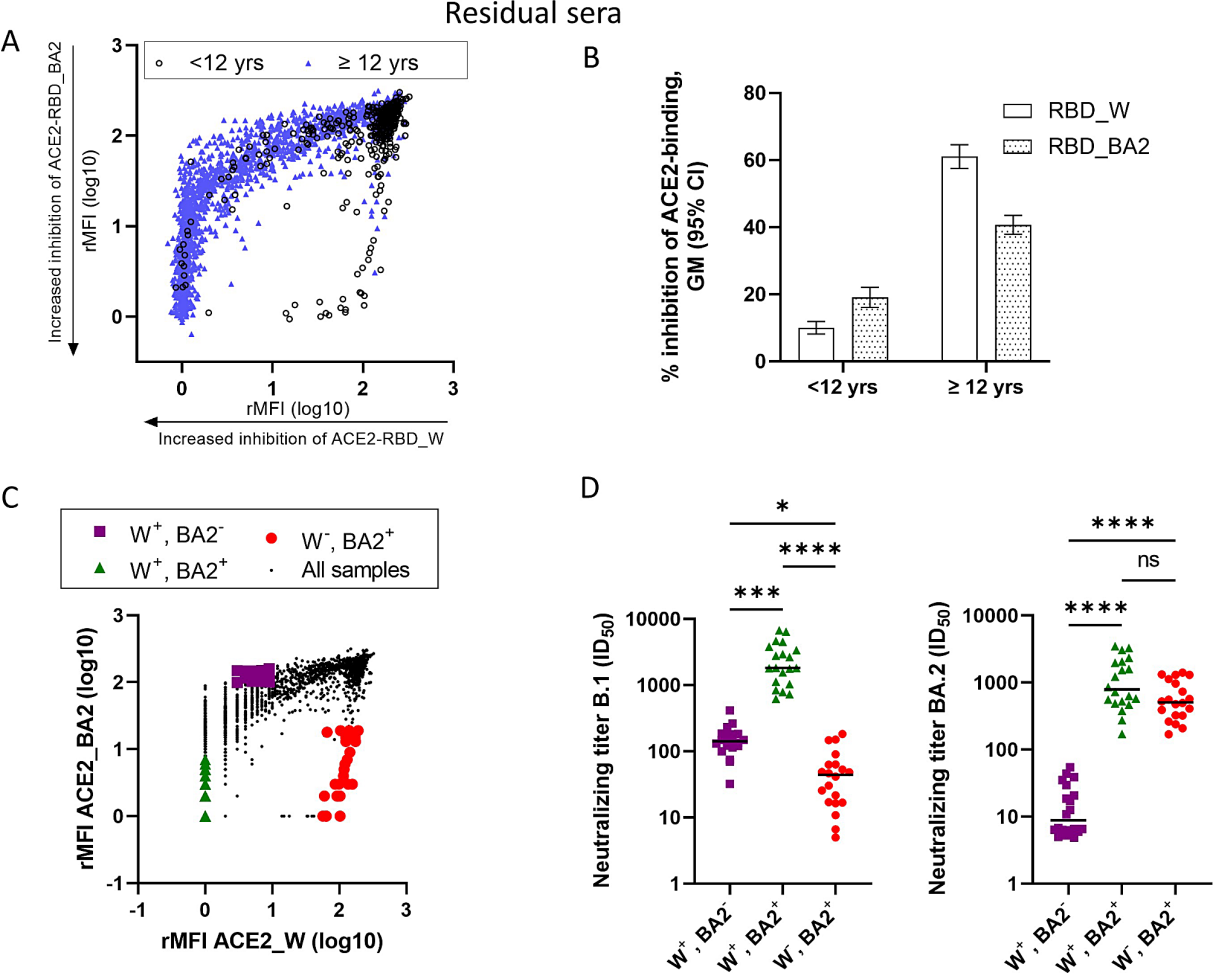
Inhibition of ACE2-RBD interaction and neutralization of B.1 and BA.2 viruses. **A**) and **B**) shows results from a bead-based assay measuring antibodies able to inhibit the interaction between ACE2, a receptor for the virus, and the SARS-CoV-2 receptor binding domain (RBD). Residual sera were tested against both Wuhan (W) and Omicron BA.2 (BA2) RBD covered beads. GM: geometric mean **C**) Distribution of three subgroups of residual sera manually chosen for neutralization assays based on their RBD-ACE2 interaction assay results: Inhibition of both W and BA.2 type interaction (W^+^BA2^+^), inhibition of W and not BA.2 (W^+^BA2^−^) and inhibition of BA.2, but not W (W^−^BA2^+^) (*n* = 20 for each group). **D**) Neutralization of B.1 (Wuhan like) and BA.2 (Omicron) viruses by subgroups explained in **C**) Dots indicate individual samples. The horizontal lines represent the medians. *p < 0.05, ***p < 0.001, ****p < 0.0001. rMFI = relative median fluorescent intensity

All residual sera were tested for the ability to inhibit binding of RBD_W/RBD_BA2 to the surface receptor ACE2 used by SARS-CoV-2 for entry into host cells (Fig. 3A and B). We observed a strong inhibition of ACE2 binding to RBD_W among individuals ≥ 12 years, while the inhibition of ACE2 binding to RBD_BA2 was 1.5 times lower (Fig. 3B). In contrast, sera from children < 12 years showed 1.9 times higher inhibition of the ACE2-RBD_BA2 interaction than the ACE2-RBD_W interaction. Overall, sera from children were less able to inhibit ACE2 binding than sera from adults.

### Neutralization assays in subsets of residual sera with different antibody profiles

To validate and extend the results from the ACE2-RBD interaction assays, three subsets of residual sera (*n* = 20 in each) were subjected to live-virus neutralization assays against isolates of the strains B.1 (Wuhan with spike D614G) and Omicron BA.2. The groups were manually selected based on their position in Fig. 3A indicating different abilities to inhibit ACE2 binding to RBD_W, RBD_BA2 or both (groups W^+^BA2^−^, W^−^BA2^+^ and W^+^BA2^+^, respectively) (Fig. 3C). As the residual sera were anonymized, the vaccination or infection status of the donors was unknown, nor was the selection based on donor age or seropositivity for anti-N antibodies. Interestingly, the W^+^BA2^+^ group had higher levels of anti-N antibodies compared to the W^+^BA2^−^ group, suggesting a higher frequency of SARS-CoV-2 infections and possibly hybrid immunity (from both vaccination and infection) (Supplementary Fig. 1A). Likewise, 60% were seropositive for anti-N antibodies in the W^+^BA2^+^ group, while in the W^+^BA2^−^ group, only 15% were seropositive for anti-N antibodies. In the W^−^BA2^+^, 90% were seropositive for anti-N antibodies, and this group was also younger in age than the other subgroups (Supplementary Fig. 1B) with a median age of 7.3 years (75% were < 12 years). The W^+^BA2^−^ and W^+^BA2^+^ groups were similar in age and consisted of mostly adults (median age 35.8 and 45.8 years, respectively). The neutralization assays confirmed that the W^+^BA.2^+^ group efficiently neutralized both B.1 and BA.2 (Fig. 2D and Supplementary Table 4). The W^+^BA2^−^ group predominantly neutralized B.1, while the W^−^BA2^+^ group efficiently neutralized BA.2 but less efficiently B.1.

### Assessing vaccination and infection history from antibody reactivity profiles

**Fig. 4:**
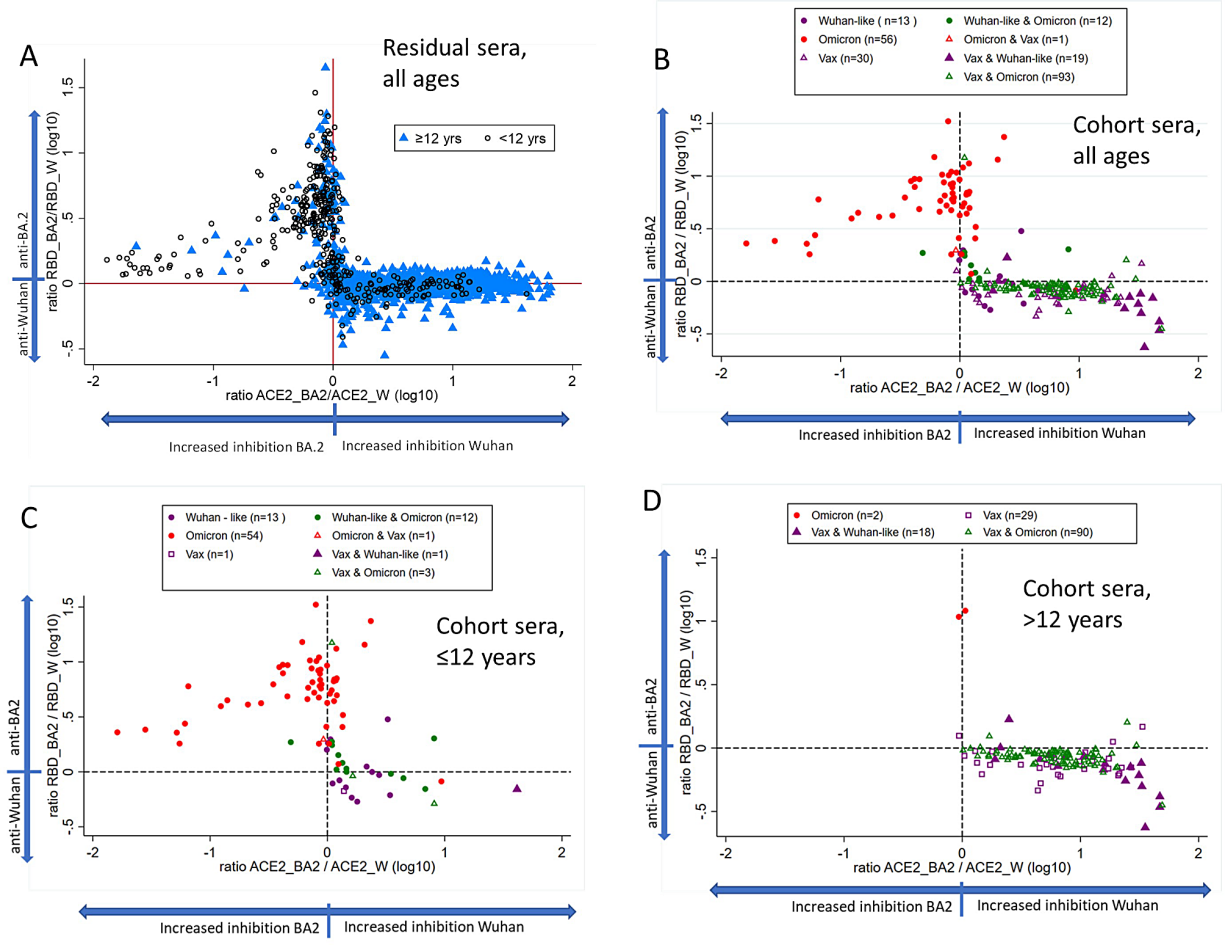
Predicting vaccination and infection history based on antibody reactivity profile. **A**) Antibody reactivity profiles of residual sera. One outlier serum (no SARS-CoV-2 antibodies) was excluded from the graph. **B**) Antibody reactivity profiles of all cohort participants with reported SARS-CoV-2 infection and COVID-19 vaccination status (*n* = 224). **C**) Antibody reactivity of cohort children ≤ 12 years (*n* = 85). **D**) Antibody reactivity profile of cohort adults > 12 years (*n* = 139). In B, C and D, Vax = vaccinated with at least one dose of COVID-19 vaccine, “Wuhan-like” indicates infection with the Wuhan, Alpha or Delta variants. Omicron refers to BA.1, BA.2 or BA.5 infection. The different combinations of vaccination and infections with order of primary exposures are indicated as follows; primary exposure to Wuhan-like antigens only (purple symbols), to Omicron antigens only (red circle), or primary exposure to Wuhan-like and subsequently Omicron antigens (green). Triangles and circles indicate primary exposure through vaccination and infection respectively

Based on different antibody reactivity patterns in residual sera from children versus older individuals as shown in Fig. 4A, we hypothesized that the antibody profiles could be used to assess vaccination and infection history. Due to the epidemiology of SARS-CoV-2 in Norway and the low vaccination coverage in children [11], a primary Omicron infection was probably the first encounter with SARS-CoV-2 for most children, leading to higher levels of antibodies against RBD_BA2 than RBD_W. Moreover, after a primary infection with Omicron, the levels of antibodies inhibiting the AC2-RBD_W interaction would be expected to be lower than in individuals with two or more doses of COVID-19 vaccine. This corresponds well with our findings in sera from children < 12 years (Fig. 4A, clustering of circles in the left upper quadrant). Many of the samples from older individuals seemed to have similar levels of antibodies against RBD_W and RBD_BA2 but giving more inhibition of ACE2 binding to RBD_W than to RBD_BA2 (Fig. 4A, clustering of triangles on the horizontal line on the right-hand side of the graph). This likely represents an antibody response induced by COVID-19 vaccines, which until September 2022 were all based on the ancestral Wuhan strain, or alternatively unvaccinated individuals infected with a Wuhan-like strain. Residual sera with an efficient inhibition of RBD_W and RBD_BA2 (mostly adult) may represent COVID-19 vaccinated individuals who were subsequently infected (Fig. 4A).

### Validation of antibody reactivity profiles using cohort sera

As the residual sera are collected anonymously, the infection and vaccination history of the donors were unknown. Thus, to test our hypothesis, we studied the antibody reactivity profile in cohort samples from families recruited in the Oslo area, including children (≤ 12 years) and adults (> 12 years), for whom vaccination status was available for all participants, and reported infection history for the vast majority (224/243, 94.2%) (Table 2). Between 23 May-13 October 2022, a subset of mothers (*n* = 80, aged 38–54 years), children (*n* = 76, aged 11–12 years), fathers (*n* = 52, aged 42–64 years) and siblings (*n* = 35, aged 8–15 years) donated blood samples (Fig. 1). The median age for the cohort children was 12 years (range 8–12 years, of which 73 (81.1%), were 11–12 years) and 47 years for the cohort adults (range 13–64 years).

**Table 2:** Infection and vaccination status of cohort participants (all), and according to age

Exposure groups	All cohort participants, *n* = 243				Cohort children (≤ 12 years), *n* = 90 (37.0%)				Cohort adults (> 12 years), *n* = 153 (63.0%)			
	Total, *n* (%)	Seropositive Wuhan method 1^a^, *n* (%)	Seropositive Wuhan & BA.2 method 2^b^, *n* (%)	Seropositive N^c^, *n* (%)	Total, *n* (%)	Seropositive Wuhan method 1, *n* (%)	Seropositive Wuhan & BA.2 method 2, *n* (%)	Seropositive N, *n* (%)	Total, *n* (%)	Seropositive Wuhan method 1, *n* (%)	Seropositive Wuhan & BA.2 method 2, *n* (%)	Seropositive N, *n* (%)
All	243 (100)	210 (86.4)	240 (98.8)	67 (27.6)	90 (100)	59 (65.6)	87 (96.7)	38 (42.2)	153 (100)	151 (98.7)	153 (100)	29 (19)
Vaccinated at any time	157 (64.6)	156 (99.4)	157 (100)	31 (19.7)	6 (6.7)	5 (83.3)	6 (100)	3 (50)	151 (98.7)	151 (100)	151 (100)	28 (18.5)
Unvaccinated	86 (35.4)	54 (62.8)	85 (98.8)	35 (41.7)	84 (93.3)	54 (64.3)	83 (98.8)	35 (41.7)	2 (1.3)	0 (0)	2 (100)	1 (50)
Infected any time^d^	194 (79.8)	165 (85.1)	193 (99.5)	60 (30.9)	84 (93.3)	57 (67.9)	83 (98.8)	36 (42.9)	110 (71.9)	108 (98.2)	110 (100)	24 (21.8)
**Times infected** ^e^												
Infected once only^d^	165 (67.9)	139 (84.2)	164 (99.4)	44 (26.7)	64 (71.1)	40 (62.5)	63 (98.4)	24 (37.5)	101 (66)	99 (98)	101 (100)	20 (19.8)
Infected twice^d^	28 (11.5)	25 (89.3)	28 (100)	16 (57.1)	20 (22.2)	17 (85)	20 (100)	12 (60)	8 (5.2)	8 (100)	8 (100)	4 (50)
**Mutually exclusive exposure groups**												
Vaccinated only^d^	30 (12.3)	30 (100)	30 (100)	3 (10)	1 (1.1)	1 (100)	1 (100)	0 (0)	29 (19)	29 (100)	29 (100)	3 (10.3)
Infected only^d^	81 (33.3)	53 (65.4)	80 (98.8)	34 (42)	79 (87.8)	53 (67.1)	78 (98.7)	33 (41.8)	2 (1.3)	0 (0)	2 (100)	1 (50)
Infected and vaccinated^d^	113 (46.5)	112 (99.1)	113 (100)	26 (23)	5 (5.6)	4 (80)	5 (100)	3 (60)	108 (70.6)	108 (100)	108 (100)	23 (21.3)
Vaccinated, infection status unknown	14 (5.8)	14 (100)	12 (85.7)	2 (14.3)	0 (0)	NA	NA	NA	14 (9.2)	14 (100)	12 (85.7)	2 (14.3)
Uninfected and unvaccinated	5 (2.1)	1 (20)	3 (60)	2 (40)	5 (5.6)	1 (20)	3 (60)	2 (40)	0 (0)	NA	NA	NA
**Order of vaccination/infection**												
Vaccinated and later infected^d^	106 (43.6)	105 (99.1)	106 (100)	19 (17.9)	2 (2.2)	1 (50)	2 (100)	2 (100)	104 (68)	104 (100)	104 (100)	17 (16.3)
Infected and later vaccinated^d^	7 (2.9)	7 (100)	7 (100)	3 (42.9)	3 (3.3)	3 (100)	3 (100)	1 (33.3)	4 (2.6)	4 (100)	4 (100)	2 (50)
**Virus type causing infection** ^**f**^												
Wuhan-like variant at any time^g^	48 (19.8)	48 (100)	48 (100)	25 (52.1)	27 (30)	27 (100)	27 (100)	17 (63)	21 (13.7)	21 (100)	21 (100)	8 (38.1)
Omicron at any time^d^	162 (66.7)	133 (82.1)	161 (99.4)	45 (27.8)	70 (77.8)	43 (61.4)	69 (98.6)	27 (38.6)	92 (60.1)	90 (97.8)	92 (100)	18 (19.6)

^a^Method 1: seropositivity was based on having antibodies against both spike and receptor binding domain (RBD) from the Wuhan variant of SARS-CoV-2.

^b^Method 2: seropositivity was based on either having antibodies against both RBD and spike from the Wuhan variant of SARS-CoV-2 (as in Method 1) or having antibodies against Omicron BA.2 RBD.

^c^N=nucleocapsid

^d^data missing for 14 adults (5.8% of all participants, 9.2% of adults)

^e^165 infected once, 28 twice and 1 three times

^f^groups not mutually exclusive, as some individuals were infected with both a Wuhan-like and an Omicron strain. Participants infected twice with the same variant were counted once

^g^no data considered missing, as PCR testing was mandatory during circulation

NA = not applicable

Overall, 79.8% (95% CI 74.2%-84.7%) of all the cohort participants had been infected at any time (Table 2). Infections were more prevalent in children (93.3% (95% CI 86.1–97.5)) compared to adults (71.9% (95% CI 64.1%-78.9%)). There was no strong national recommendation for children under 12 years to get vaccinated against COVID-19 during 2021/2022 and only 6.7% (95% CI 2.5%-13.9%) of the NorFlu children (aged 11–12 years at sampling) were vaccinated (Table 2). The COVID-19 vaccination rate was very high among the adults (98.7% (95% CI 95.4%-99.8%)). Most of the children were infected only (87.8% (95% CI 79.2%-93.7%)), while most of the cohort adults were vaccinated and later infected (68.0% (95% CI 60%-75.3%)) (Table 2). Infections were mainly caused by Omicron (Table 2). Estimated seroprevalence based on cohort samples was similar to the estimates from residual sera, both overall (99.5% (95% CrI 98.0–100%)) and for children and adults (Supplementary Table 5). As for residual sera, the seroprevalence estimate was considerably lower in children when only including antibodies against the ancestral Wuhan variant (method 1).

For participants with reported SARS-CoV-2 infection and COVID-19 vaccination status, i.e., infected and vaccinated, infected only, or vaccinated only (*n* = 224), we explored the antibody profiles as previously performed for the residual sera, and found the overall pattern was similar for the residual sera and the cohort sera (Fig. 4B).

The cohort children’s samples that were primed with Omicron infection, showed higher antibody levels against RBD_BA2 (red circles, Fig. 4C) than the ones that were primed with the vaccine or a Wuhan-like variant (purple and green symbols). These sera also shown higher inhibition of ACE2-RBD_2 interaction than ACE2-RBD_W interaction. As mentioned, most of the cohort adults were primed with a COVID-19 vaccine before SARS-CoV-2 infection. Their antibody profile resembled the antibody profile of most of the adult residual sera with higher levels against RBD_W than RBD_BA2, and greater inhibition of ACE2 binding to RBD_W than RBD_BA2 (lower right quadrant, Fig. 4D). Cohort children who had been primed by vaccination or with infection of a Wuhan-like variant, responded more like cohort adults (Fig. 4C and D). Individuals first vaccinated and then infected with Wuhan-like variant seemed to have higher levels of anti-RBD_W antibodies and better inhibition of ACE2 binding to RBD_W (purple triangles, Fig. 4D) than individuals vaccinated and later infected with an Omicron variant (green triangles, Fig. 4D). Likewise, two adult individuals who were unvaccinated, but infected with Omicron (red circles, Fig. 4D), presented with a profile similar to the unvaccinated children primary infected with Omicron.

### Anti-N antibodies in cohort samples

**Fig. 5:**
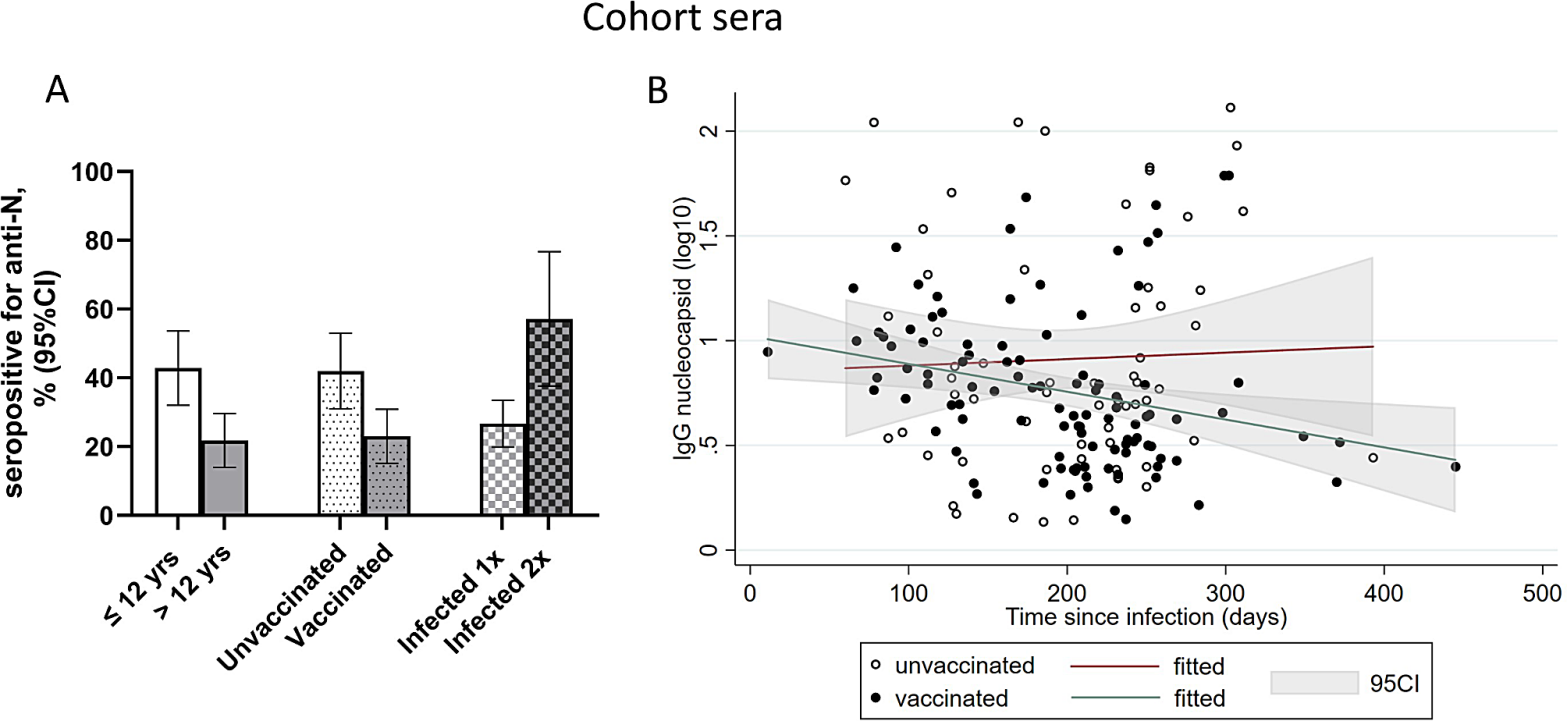
Seropositivity and waning of anti-N antibodies in cohort samples with confirmed infection/vaccination status. **A**) Percent of cohort samples seropositive for antibodies against N according to age, vaccination status and times infected. Only cases infected at least once are included. ≤12 years (*n* = 84), > 12 years (*n* = 110), unvaccinated (*n* = 81), vaccinated (*n* = 113), infected 1x (*n* = 165), infected 2x (*n* = 28). **B**) Waning of anti-N antibodies in cohort samples from vaccinated (*n* = 103) and unvaccinated (*n* = 62) individuals, all who reported to be infected once. The graph shows a proxy of waning as one individual contribute with one sample only. Dots represent individual values. The green line with grey area shows the linear regression of antibody levels with 95% CI for the slope in vaccinated individuals. The red line with grey areas shows the corresponding results from unvaccinated individuals

Next, we determined the antibody response against N among the cohort sera with confirmed infection (Fig. 5), to explore how reliable anti-N was as a marker of infection. The percentage of anti-N seropositive samples was higher in the infected children ≤ 12 years (42.9% (95% CI 32.1%-54.1%)) than in the older infected participants (21.8% (95% CI 14.5% -30.7%) (Fig. 5A). Seropositivity for anti-N antibodies among infected individuals was also higher among those who were unvaccinated (42.0% (95% CI 31.1-53.5%)), primarily children) compared to those who were infected and vaccinated (23.0% (95% CI 15.6-31.9%)) and was higher after two infections (57.1% (95% CI 37.2%-75.5%)) than one (26.7% (95% CI 20.1%-34.1%)) (Fig. 5A). Notably, 42.9% of individuals with confirmed COVID-19 did not develop antibodies to N, even after being infected twice.

The overall anti-N seropositivity, irrespective of infection/ vaccination status, amongst all the cohort children (*n* = 90) was 42.2% (95% CI 31.9%-53.1%) and 19.0% (95% CI 13.1-26.1%) for all the cohort adults (*n* = 153) (Table 2).

Some participants (5 children) did not have a record of vaccination, nor report having had an infection. Their antibody profile nevertheless suggested they had been exposed to Omicron (Supplementary Fig. 2), and furthermore 60% had anti-N antibodies (Table 2). The profile of the participants who were all vaccinated, but for whom no Omicron infection history was available (*n* = 14) had an antibody profile suggesting priming with a Wuhan-like strain (Supplementary Fig. 2).

To study if vaccination influenced the duration and prevalence of anti-N antibodies after infection, we plotted levels of anti-N antibodies versus time since infection according to vaccination status among cohort samples (Fig. 5B). This will give a proxy of waning as participants only contribute with a measurement from one time point each. Among individuals that were infected once with SARS-CoV-2, antibody waning was faster in the vaccinated than unvaccinated cases (*p* = 0.034). The antibody levels (GM rMFI) for the infected, unvaccinated group were higher than for the infected, vaccinated group (8.16 (95% CI 5.89–11.3) and 5.79 (95% CI 4.90–6.84), respectively), though not statistically significant (*p* = 0.235).

### Neutralization of newer SARS-CoV-2 variants by subsets of residual sera

**Fig. 6:**
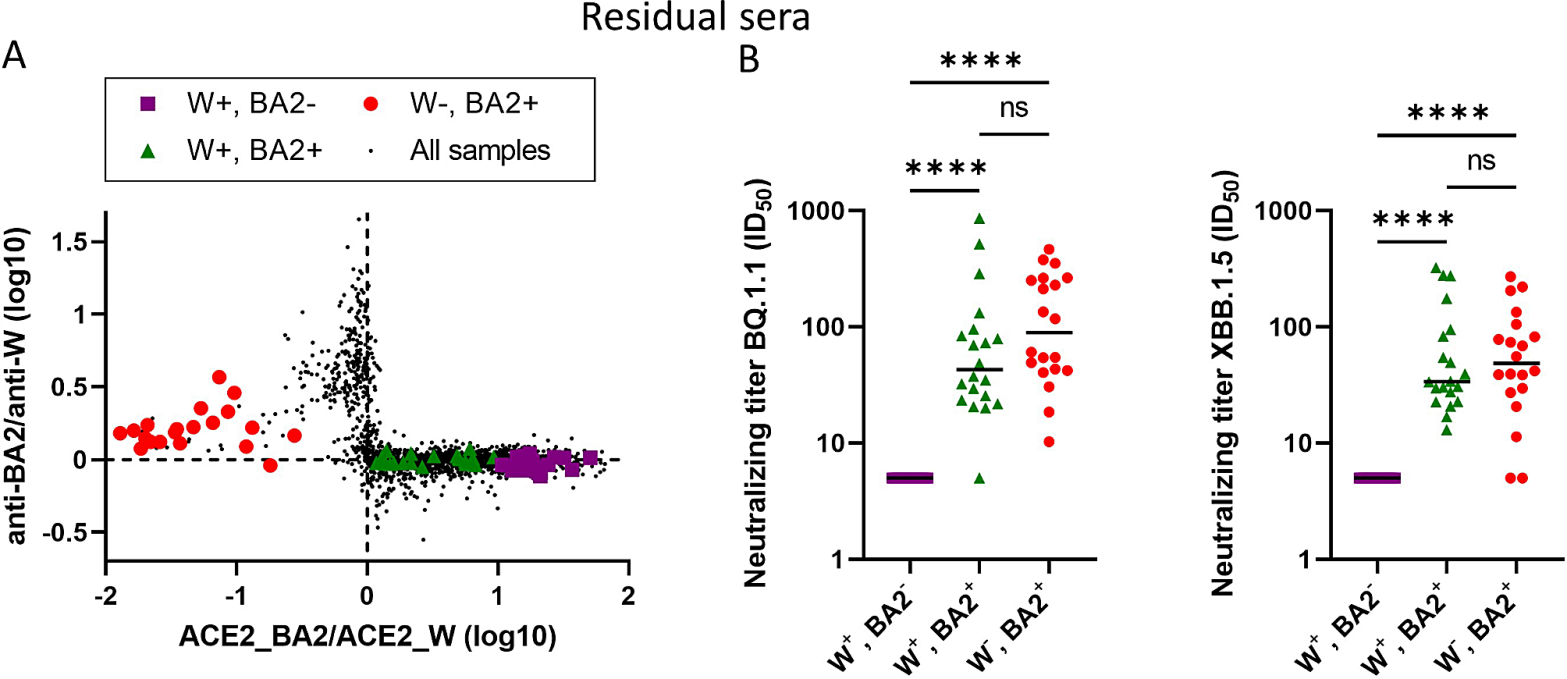
Hybrid immunity provides neutralization of newer SARS-CoV-2 variants. **A**) Antibody reactivity pattern based on anti-RBD levels and inhibition of RBD-ACE2 interactions in subgroups of residual sera are shown. RBD was of Wuhan (W) or Omicron BA.2 (BA2) type. Three subgroups of sera with different patterns are indicated (see also Fig. 3A); group W^+^BA2^+^ represents sera with a profile of hybrid immunity. **B**) Neutralization of Omicron BQ.1.1. and XBB1.5 variants by subgroups (*n* = 20 for each group). Dots indicate individual samples. The horizontal line represents the median. ****p < 0.0001. ns = not significant

While our results suggest that almost all Norwegians had antibodies towards SARS-CoV-2 in the late summer of 2022, it was unclear how well these antibodies protected against newer variants of the virus. We therefore tested the previously introduced subgroups of residual sera with the main antibody reactivity profiles in live virus neutralization assays against the Omicron variants BQ.1.1 (subvariant of BA.5) and XBB.1.5 (subvariant of BA.2), not present in Norway until late 2022. The W^+^BA2^−^, W^+^BA2^+^ and W^−^BA2^+^ subgroups were all placed within the expected antibody reactivity profiles as established with the cohort samples (Fig. 6A). When these subgroups were tested against the newer Omicron variants, the W^+^BA2^+^ and W^−^BA2^+^ groups both neutralized the BQ.1.1 and XBB.1.5 strains (Fig. 6B), although neutralizing titers were lower than against BA.2 (Supplementary Table 4). The W^−^BA2^+^ group had the highest level of neutralization against the newer strains, although the difference was not significant compared to the W^+^BA2^+^ group (Fig. 6B). The W^+^BA2^−^ group showed no neutralization of BQ.1.1 and XBB.1.5.

## Discussion

By the late summer of 2022, the seroprevalence estimate of SARS-CoV-2 from vaccination and/or infection was found to be 99.1% (95% CrI 97.0%–100.0%) in Norway, according to this nationwide cross-sectional collection of residual sera from Norwegian laboratories. By characterizing antibody reactivity against RBD_W and RBD_BA2, we observed distinct antibody profiles indicative of past infection and vaccination history (Fig. 4), supported by anti-N antibody responses. Since children < 12 years (generally unvaccinated) and older individuals (91% vaccinated ≥ 16 years) had distinct profiles, we hypothesized that differential reactivity against RBD_W and RBD_BA.2 may help to discriminate between vaccination with ancestral Wuhan-based mRNA vaccines, primary infection with Omicron variants, and hybrid immunity. This was confirmed by analyzing sera from a population-based cohort (NorFlu) with registered vaccination and/or reported infection history (Fig. 4). Younger, mostly unvaccinated individuals had a higher reactivity against RBD_BA2, while older, vaccinated individuals had higher reactivity against RBD_W. Consequently, widely used diagnostic assays, which only measure antibodies to RBD_W or spike_W, are likely to underestimate the true seroprevalence, especially in populations with primary infection with Omicron variants.

### Seroprevalence estimates

In August 2021, the estimated seroprevalence from infection and/or vaccination was 62.6% (95% CrI 60.1%-65.2%) for Norway [14]. By the late summer of 2022, the national estimated seroprevalence had increased by more than 35 percentage points (Fig. 2C), probably due to a combination of both increases in COVID-19 vaccination and cases [11]. Throughout 2021 and 2022, the COVID-19 vaccination campaign continued and was also expanded to younger age groups [16, 29], increasing the national vaccine coverage for ≥ 1 dose from 58% to 78% [11, 30]. As of mid-July 2022, confirmed COVID-19 cases was recorded for 26.5% of the Norwegian population [11]. However, the actual number of infections was most likely much higher, which is supported by the present study estimating a very high seroprevalence in the largely unvaccinated child population and the high percentage of reported infection among the cohort participants (93.3% for children, 71.9% for adults). The emergence of Delta and Omicron variants also increased the frequency of infections in previously vaccinated individuals (breakthrough infections) [12]. In a Norwegian survey of symptoms, 40–70% of responders aged ≥ 16 years reported at least one episode of COVID-19 by June 2022, and the percentage was higher for younger than older individuals [31]. Questionnaire data from the nationwide Norwegian Mother, Father and Child study (MoBa) suggested that > 70% of children aged 12–15 years had been infected by May 2022, and by June 2022, 56% of adults > 18 years had been infected at least once [31].

The Omicron BA.2 variant dominated in Norway between February and mid-June 2022 and caused the largest increase in number of infections since the start of the pandemic until sample collection in the late summer of 2022 (Fig. 1) [11]. Since we observed that some individuals, primarily children, had antibodies against Omicron RBD_BA2 and not RBD_W (Fig. 2A), our previous strategy of only determining antibodies against the Wuhan variant would underestimate the SARS-CoV-2 seroprevalence in Norway. Our updated method (method 2), which also includes antibodies against Omicron variant BA.2, gave only a small increase in the estimated seroprevalence for individuals > 12 years and the national seroprevalence estimate. However, in samples from children < 12 years, the updated method increased the seroprevalence estimate with 14 percentage points, likely reflecting a high degree of primary Omicron infections among children, which is also supported by the high seropositivity of anti-N antibodies and relatively high correlation between anti-RBD and anti-N in these sera. In August 2021, the SARS-CoV-2 seroprevalence estimate in children < 12 years was 12.5% (95% CrI 9.3–16.1%) [14]; thus, the increase in the seroprevalence estimate between August 2021 and the late summer of 2022 was 85 percentage points in this age group. Since COVID-19 vaccines used in Norway were not approved for use in children under 5 years [32] and only 2% of children aged 5–11 were vaccinated by mid-July 2022 [11], the majority of this increase was caused by SARS-CoV-2 infections. Our finding corresponds well with a global meta-analysis, suggesting that 50–70% of children were immunologically naïve for SARS-CoV-2 prior to the first Omicron wave [33]. Similarly, cohort data indicate that most infections in children were caused by Omicron.

In Finland, another Nordic country, the seroprevalence based on anti-spike IgG antibodies, was 98% (95% CI 76%-100%) in the third quarter of 2022 [34], similar to our findings. The proportion of adults with hybrid immunity was lower in Finland (55% (95% CI 23%-83%)) than the proportion observed in our adult cohort (70.6% with a record of both infection and vaccination), while the Finnish seroprevalence based on anti-N IgG was higher (55% (95% CI 23%-83%)) than the proportion of anti-N seropositive cohort adults presented here (19%). In a systematic review and meta-analysis, the overall seroprevalence from infection and vaccination in European high-income countries was 95.9% (95% CI 92.3% − 97.8%) in March 2022 [35].

### Antibodies against N

COVID-19 vaccines have been shown to protect well against severe disease, but less well against transmission and infection, particularly as the virus has evolved [36]. Due to extensive immune evasion seen with Omicron, the Wuhan spike-based COVID-19 mRNA vaccines used in the national vaccination program in Norway in 2020–2022 had limited effect on preventing transmission [12]. Nevertheless, residual sera from the largely unvaccinated child population were more likely to have antibodies against N, than sera from the highly vaccinated older population. Likewise, a higher proportion of the mainly unvaccinated cohort children reported infection (93%) compared to the vaccinated adults (72% reported infections), suggesting protection from COVID-19 vaccination. The proportion of anti-N seropositive children was comparable, but slightly higher in the residual sera compared to the cohort sera. This may be due to the residual sera coming from individuals with more comorbidities (collected at medical laboratories) while the cohort individuals may have a healthy-person bias. This was also the case for the adult sera, although the difference was larger (35% vs. 19%, respectively), which may reflect a healthier, younger, and more vaccinated cohort population.

Irrespectively, the percentages of residual sera with anti-N-antibodies were higher in all age groups in the late summer of 2022 compared to August 2021 (Fig. 2), in agreement with the increase in confirmed COVID-19 cases [11]. The increase in anti-N positivity was lower in older age groups, suggesting less SARS-CoV-2 infections, in accordance with the previously mentioned Norwegian symptom survey [31]. In a US study, conducted post-Omicron, the overall seroprevalence of anti-N antibodies increased from 33.5% in September 2021 to 57.7% in February 2022 [37]. The increase was even more pronounced for children, from 44.2 to 75.2%, whereas the seroprevalence increased the least in persons aged ≥ 65 years. In a Portuguese study, based on residual sera collected between April and June 2022 and measuring anti-N and anti-S antibodies, a seroprevalence of 95.5% from infection or vaccination was found [38]. Overall, 27.3% of sera were positive for anti-N antibodies. Seroprevalence of anti-N antibodies was highest among children aged 0–4 years (39.2%) and lowest among adults ≥ 70 years (17.3%). These findings are in line with the results presented here.

### Anti-N antibodies as a marker of SARS-CoV-2 infection

Although children were more likely than older individuals to have anti-N antibodies, only 50% of residual sera from this age group were seropositive, while the estimated seroprevalence based on spike and RBD antibodies was close to 100%. Previously, we have observed that 31.9% of seropositive (spike and RBD antibodies) samples from an unvaccinated child population lacked antibodies against N, implying that these antibodies are unreliable indicators of past SARS-CoV-2 infection in a population [14]. In the present study, 42.9% of samples from cohort participants who had reported being infected twice were seronegative for anti-N antibodies. Others have reported that antibody levels against N are correlated with severity [39] and that children are less likely to develop anti-N antibodies or develop lower levels of these antibodies than adults [40]. We observed that after infection, cohort children were more likely to be seropositive for anti-N antibodies than the cohort adults. We also observed that after SARS-CoV-2 infection, unvaccinated individuals were more likely to develop anti-N-antibodies than individuals vaccinated prior to the infection, as observed in several studies [6, 7, 41, 42](Fig. 5). Few cohort children were vaccinated, which could explain why more children developed anti-N antibodies. A Japanese study from 2022 found that approximately 80% of individuals with self-reported COVID-19 (90% were vaccinated) lacked anti-N antibodies [43]. Furthermore, antibodies against N wane faster than antibodies against spike and RBD [4, 44]. Here we estimated that anti-N antibody waning was faster in vaccinated compared to unvaccinated cohort participants, likely contributing to the lower prevalence of anti-N antibodies in the highly vaccinated adult population. This may be due to more rapid viral clearance in the vaccinated [7] and therefore a more limited and short-lived immune activation [45, 46]. Consequently, anti-N seropositivity cannot be used to determine the number of SARS-CoV-2 infections in a highly vaccinated, adult population, nor the number of times an individual has been infected. Therefore, we did not estimate a seroprevalence of SARS-CoV-2 infection based on anti-N antibodies. Further, as we did not take anti N-antibody waning, nor the potential for less seroconversion in vaccinated individuals into account, the numbers of true infections may have been higher than the proportion of anti-N positive samples reported here. Indeed, the cohort data displays the discrepancies between the proportions with reported infections and the proportion seropositive for anti-N (Table 2). Nevertheless, the increase in percentage of anti-N-seropositive samples from 2021 to 2022 supports an increase in infections for all ages. Additionally, the high proportion of infections among cohort adults (79.1%, of which 68% were vaccinated, then infected) as well as the hybrid immunity profile displayed by a large proportion of the residual sera indicates a high degree of subsequent infection also among vaccinated adults. The differences in anti-N-positive samples between age groups with similar vaccination coverage could indicate differences in exposure, severity of disease, or risk-taking behavior.

### Antibody reactivity profiles of residual sera validated by cohort sera

Based on the levels of RBD_W and RBD_BA2 binding antibodies and the inhibition of RBD-ACE2 interaction of each residual sera, we generated antibody reactivity profiles displaying a difference in antibody responses between children < 12 years and older individuals (Fig. 4). The differences in RBD-ACE2 interactions were confirmed by neutralization assays (Fig. 3). Sera from children were more likely to have high levels of antibodies against RBD_BA2 and to preferentially neutralize Omicron BA.2 virus (W^−^BA2^+^ group), while sera from adults were more likely to have high antibody levels against RBD_W and neutralize the Wuhan variant (W^+^BA2^−^ and W^+^BA2^+^ groups) (Fig. 3). The COVID-19 vaccines used in Norway were based on the ancestral Wuhan variant of SARS-CoV-2 until after the time of sampling. Antibody levels have been reported to be lower after primary infection than after two doses of COVID-19 vaccination or after breakthrough infections [47–49]. The low COVID-19 vaccine coverage, combined with high anti-N antibody seropositivity and higher antibody levels against RBD_BA2 than RBD_W in residual sera from children < 12 years, also point to primary infections with Omicron in this age group. This was largely confirmed by sera from cohort participants with a primary Omicron infection displaying a similar antibody reactivity pattern (Fig. 4). Moreover, sera from cohort adults could be used to define an antibody reactivity pattern of COVID-19 vaccinated individuals, as well as a pattern of hybrid immunity.

### Neutralization of newer viral variants in sera displaying hybrid immunity and potential immune imprinting

Previous studies have indicated that COVID-19 vaccination with spike from the Wuhan variant or infection with a pre-Omicron variant may limit the induction of neutralizing antibodies when infected with an Omicron variant [50]. The phenomenon is referred to as immune imprinting, i.e., that the first exposure to a virus leaves an immunological “imprint”. Thus, subsequent exposures to variants of the virus will predominantly boost antibodies targeting epitopes shared between the first virus and later variants [51, 52]. In our study we observed that initial exposure to Wuhan determined the subsequent antibody profile (Fig. 4). For instance, cohort participants first vaccinated with a Wuhan-based vaccine maintained their antibody profile with a higher reactivity to RBD_W after breakthrough infection with an Omicron variant. These observations are in accordance with immune imprinting after COVID-19 vaccination, and that subsequent infections induced a high degree of back-boosting of antibodies specific for the original Wuhan strain [50].

However, our neutralization assays using residual sera with a profile suggesting priming with Wuhan-based COVID-19 vaccines and subsequent Omicron infection (W^+^BA2^+^ group) still induced neutralizing antibodies against BA.2. Moreover, their neutralizing antibody titers against BA.2 were comparable to individuals hypothesized to be primed with an Omicron infection (predominantly children < 12 years) (W^−^BA2^+^ group) (Fig. 3). Interestingly, 60% of the W^+^BA2^+^ group had detectable levels of anti-N antibodies, supporting the hypothesis of hybrid immunity in this group. Consequently, the potential immune imprinting suggested by the antibody reactivity profiles does not seem to negatively impact the ability to induce neutralizing antibodies against new variants after infection.

In fact, we also observed that the W^+^BA2^+^ group that efficiently neutralized both B.1 and BA.2 virus, also more efficiently neutralized the BQ.1.1 and XBB.1.5, SARS-CoV-2 variants that emerged after the sera was collected, compared to sera from the W^+^BA2^−^ group with only 15% anti-N seropositivity, indicating an antibody response predominantly from vaccination (Fig. 6). The present results showing an association between high seropositivity of anti-N and neutralizing activity against Omicron variants XBB.1.5 and BQ.1.1 suggest that breakthrough infections lead to induction of antibodies capable of cross-reacting with future variants. These observations correspond to previous studies showing that serum sampled after breakthrough infections with Omicron are better at neutralizing various Omicron variants than sera from individuals only vaccinated with the Wuhan ancestral variant and that Omicron breakthrough infection alleviates potential imprinting from vaccination with Wuhan-type COVID-19 vaccines [21, 53]. Considering that the majority of adult Norwegians in all probability had undergone both vaccination and infection (70.6% of cohort adults) and acquired hybrid immunity in the late summer of 2022, the Norwegian population most likely had a significant degree of protection also against new emerging Omicron variants. It is also important to note that although titers of neutralizing antibodies against Omicron variants were lower in sera from individuals that we hypothesize were vaccinated only, studies have shown that Wuhan-based COVID-19 vaccines are protective against severe disease caused by Omicron variants [54, 55].

### Strengths and limitations

A limitation of this study is that the residual sera came from medical laboratories, which could introduce a selection bias towards individuals with more morbidity and more active healthcare-seeking behavior. Conversely, population-based cohort studies where study subjects are invited to participate, could lead to healthy-person bias and exclusion of certain groups [56]. However, obtaining results from both these types of sera and finding comparable results, strengthens our study. We did not consider antibody waning, but antibodies against spike and RBD have been shown to be long-lasting [3] and the high seroprevalence estimates indicate that waning was not a problem in the present study. The residual sera study population was slightly younger than the Norwegian population, but this has also been the case for our previous SARS-CoV-2 seroprevalence studies [8, 14], thus estimates are comparable over time.

For the cohort participants, there may be some misclassification of virus variants during periods of variant replacement, as virus type was assigned based on the dates of infection. Moreover, some of the cohort infections (mostly Omicron) were self-reported after mandatory testing was stopped. Also, some infections, particularly amongst the vaccinated adults, may have remained undetected depending on testing behavior, especially after mandatory testing (including of contacts), was abolished. Asymptomatic infections may be less accounted for, and we cannot exclude recall bias. Another limitation is that cohort participants were restricted to families from a city, with children under 15 years, which may have influenced transmission dynamics, and therefore prevalence estimates. Also, young adults aged 16–37 years and older adults (> 65 years) are not represented among the cohort participants. The timing of sampling was also somewhat different for the two study populations, the residual sera were collected primarily in August 2022, while the cohort samples were collected between May and October 2022 (Fig. 1). Nevertheless, we were able to obtain similar seroprevalence estimates using both study populations and confirm the antibody profiles from the residual sera with cohort samples, strengthening our findings. In the W^+^BA2^+^ group, which we have interpreted as having hybrid immunity, there could be individuals with Wuhan infection and not COVID-19 vaccination as vaccination coverage was 87% in the age group 35–44 years in mid-July 2022 (median age 35 years in W^+^BA2^+^ group) [11].

## Conclusions

In the late summer of 2022, nearly the entire Norwegian population had antibodies against SARS-CoV-2 from vaccination and/or infection. The high seroprevalence estimate in residual sera from children < 12 years, and high proportion of reported infections among cohort children, implies a very high degree of SARS-CoV-2 infections among children in general. The antibody profiles observed in residual sera indicate that children mostly had experienced a primary Omicron infection, while hybrid immunity was common among adults, as supported by cohort data. Measurements of anti-N antibodies show increases of SARS-CoV-2 infections in all age groups since 2021. However, due to limitations in the utility of anti-N-antibodies as a proxy for infection, estimation of the extent of infections in the adult Norwegian population is challenging. Furthermore, we have shown that with the continued emergence of newer and antigenically different variants of SARS-CoV-2, the antibody landscape becomes more complicated and that some immunity and exposures may be overlooked if the serology assays are less suited for newer variants. Finally, immune imprinting from ancestral Wuhan-based COVID-19 vaccines does not seem to impact the ability to gain immunity against drift variants.

### Electronic supplementary material

Below is the link to the electronic supplementary material.

Supplementary Material 1

Supplementary Material 2

Supplementary Material 3

## Data Availability

The data that support the findings of this study regarding the residual sera are available from the corresponding author upon reasonable request. The NorFlu cohort study consist of sensitive information on an individual level. Due to protection of privacy and restrictions from the Norwegian Data Inspectorate and the Regional Committee for Medical and Health Research Ethics, the data are not publicly available.

## Abbreviations

COVID-19: Coronavirus disease 2019
CI: Confidence intervals
CrI: Credible intervals
GM: Geometric mean
GMC: Geometric mean concentration
N: Nucleocapsid
MSIS: Norwegian Surveillance System for Communicable Diseases
MoBa: The Norwegian Mother, Father and Child Cohort Study
RBD: Receptor binding domain
RBD_W: RBD from the Wuhan variant
RBD_BA2: RBD from the Omicron BA.2 variant
rMFI: Relative median fluorescent intensity
SARS-CoV-2: Severe acute respiratory syndrome coronavirus 2
Spike_W: Spike from the Wuhan variant
SYSVAK: Norwegian Immunisation Registry
