# Supplementary material for: Characterization of the SARS-CoV-2 antibody landscape in Norway in the late summer of 2022: high seroprevalence in all age groups with patterns of primary Omicron infection in children and hybrid immunity in adults
Source: BMC Infect Dis. 2024 Aug 20;24:841. doi: 10.1186/s12879-024-09670-w (PMC11334563; doi:10.1186/s12879-024-09670-w)
Supplement: Supplementary file 1 — Supplementary Material 1 [file 12879_2024_9670_MOESM1_ESM.pptx]

## Slide 1
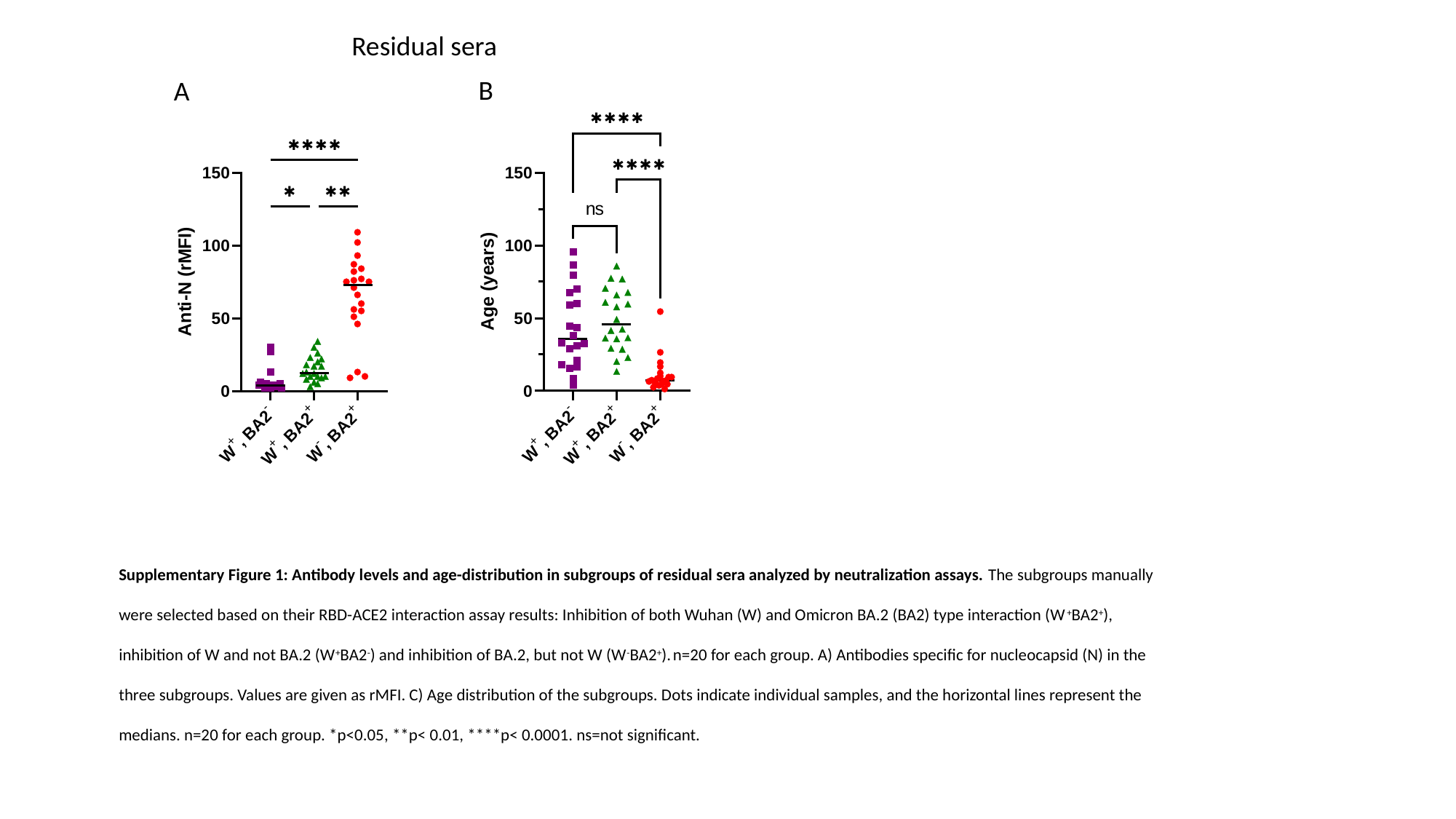

Residual sera
B
A
Supplementary Figure 1: Antibody levels and age-distribution in subgroups of residual sera analyzed by neutralization assays. The subgroups manually were selected based on their RBD-ACE2 interaction assay results: Inhibition of both Wuhan (W) and Omicron BA.2 (BA2) type interaction (W+BA2+), inhibition of W and not BA.2 (W+BA2-) and inhibition of BA.2, but not W (W-BA2+). n=20 for each group. A) Antibodies specific for nucleocapsid (N) in the three subgroups. Values are given as rMFI. C) Age distribution of the subgroups. Dots indicate individual samples, and the horizontal lines represent the medians. n=20 for each group. *p<0.05, **p< 0.01, ****p< 0.0001. ns=not significant.
