# Supplementary material for: Characterization of the SARS-CoV-2 antibody landscape in Norway in the late summer of 2022: high seroprevalence in all age groups with patterns of primary Omicron infection in children and hybrid immunity in adults
Source: BMC Infect Dis. 2024 Aug 20;24:841. doi: 10.1186/s12879-024-09670-w (PMC11334563; doi:10.1186/s12879-024-09670-w)
Supplement: Supplementary file 2 — Supplementary Material 2 [file 12879_2024_9670_MOESM2_ESM.pptx]

## Slide 1
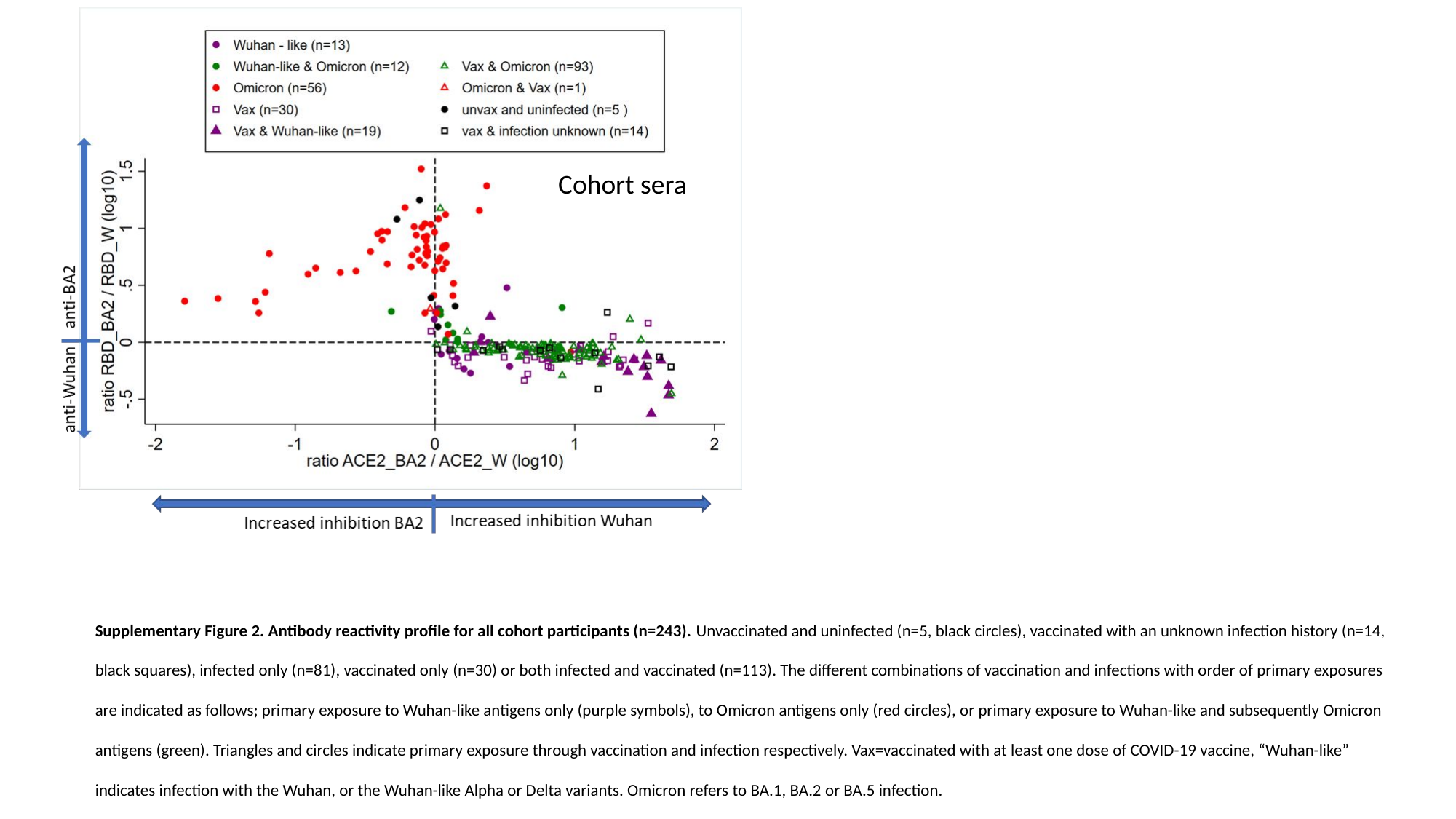

Cohort sera
Supplementary Figure 2. Antibody reactivity profile for all cohort participants (n=243). Unvaccinated and uninfected (n=5, black circles), vaccinated with an unknown infection history (n=14, black squares), infected only (n=81), vaccinated only (n=30) or both infected and vaccinated (n=113). The different combinations of vaccination and infections with order of primary exposures are indicated as follows; primary exposure to Wuhan-like antigens only (purple symbols), to Omicron antigens only (red circles), or primary exposure to Wuhan-like and subsequently Omicron antigens (green). Triangles and circles indicate primary exposure through vaccination and infection respectively. Vax=vaccinated with at least one dose of COVID-19 vaccine, “Wuhan-like” indicates infection with the Wuhan, or the Wuhan-like Alpha or Delta variants. Omicron refers to BA.1, BA.2 or BA.5 infection.
