# Supplementary material for: Characterization of the SARS-CoV-2 antibody landscape in Norway in the late summer of 2022: high seroprevalence in all age groups with patterns of primary Omicron infection in children and hybrid immunity in adults
Source: BMC Infect Dis. 2024 Aug 20;24:841. doi: 10.1186/s12879-024-09670-w (PMC11334563; doi:10.1186/s12879-024-09670-w)
Supplement: Supplementary file 3 — Supplementary Material 3 [file 12879_2024_9670_MOESM3_ESM.docx]

| **Supplementary Table 1 Number of samples and seroprevalence estimates by week number** | | | | | | | | |  |
| --- | --- | --- | --- | --- | --- | --- | --- | --- | --- |
| **Week (W)**  **no^a^** | **Number of samples tested (%)** | **Positive samples, method 1^b^**  **(Wuhan antibodies)** | **Positive samples, % (95% CI), method 1** | **Seroprevalence estimate, % (95% credible interval), method 1** | | **Positive samples, method 2^c^ (Wuhan & BA.2 antibodies)** | **Positive samples, % (95% CI), method 2** | **Seroprevalence estimate, % (95% credible interval), method 2** |  |
| W20 | 1 (0.1%) | 1 | 100.0 (2.5 - 100.0) | | 66.6 (16.9 - 98.7) | 1 | 100.0 ( 2.5 - 100.0) | 90.5 (55.6 - 99.9) |  |
| W21 | 5 (0.3%) | 4 | 80.0 (28.4 - 99.5) | | 73.2 (36.7 - 97.5) | 4 | 80.0 (28.4 - 99.5) | 92.9 (73.9 - 99.9) |  |
| W29 | 13 (0.7%) | 13 | 100.0 (75.3 - 100.0) | | 93.5 (77.5 - 99.8) | 13 | 100.0 (75.3 - 100.0) | 98.8 (94.5 - 100.0) |  |
| W30 | 266 (13.9%) | 248 | 93.2 (89.5 - 95.9) | | 95.6 (91.9 - 98.9) | 254 | 95.5 (92.3 - 97.6) | 98.8 (96.1 - 100.0) |  |
| W31 | 661 (34.5%) | 618 | 93.5 (91.3 - 95.3) | | 96.1 (93.6 - 98.8) | 642 | 97.1 (95.5 - 98.3) | 99.2 (97.2 - 100.0) |  |
| W32 | 403 (21.1%) | 374 | 92.8 (89.8 - 95.1) | | 95.3 (92.2 - 98.5) | 389 | 96.5 (94.2 - 98.1) | 99.0 (96.5 - 100.0) |  |
| W33 | 242 (12.6%) | 219 | 90.5 (86.1 - 93.9) | | 92.8 (88.4 - 96.8) | 237 | 97.9 (95.2 - 99.3) | 99.1 (96.7 - 100.0) |  |
| W34 | 164 (8.6%) | 147 | 89.6 (83.9 - 93.8) | | 91.8 (86.2 - 96.6) | 155 | 94.5 (89.8 - 97.5) | 98.2 (94.2 - 100.0) |  |
| W35 | 35 (1.8%) | 33 | 94.3 (80.8 - 99.3) | | 94.0 (83.3 - 99.7) | 34 | 97.1 (85.1 - 99.9) | 98.8 (95.2 - 100.0) |  |
| W36 | 1 (0.1%) | 1 | 100.0 (2.5 - 100.0) | | 65.2 (14.5 - 98.5) | 1 | 100.0 (2.5 - 100.0) | 90.8 (56.8 - 99.9) |  |
| Missing | 123 (6.4%) | 118 | 95.9 (90.8 - 98.7) | | 97.4 (92.8 - 99.9) | 122 | 99.2 (95.6 - 100.0) | 99.5 (98.1 - 100.0) |  |
|  | | | | | | | | |  |

^a^W 20-21: May 2022, W29-30: July 2022, W31-35: August 2023, W35-36: September 2022. ^b^Method 1: seropositivity was based on having antibodies against both spike and receptor binding domain (RBD) from the Wuhan variant of SARS-CoV-2. ^c^Method 2: seropositivity was based on either having antibodies against both RBD and spike from the Wuhan variant of SARS-CoV-2 (as in Method 1) or having antibodies against RBD from Omicron BA.2.

**Supplementary Table 2. Seroprevalence estimates for individuals under and over 12 years of age, 2022**

|  | | | | | | | |  |
| --- | --- | --- | --- | --- | --- | --- | --- | --- |
| **Age (years)** | **Number of samples tested (%)** | **Positive samples, method 1^a^ (Wuhan antibodies)** | **Positive samples, % (95% CI), method 1** | **Estimated seroprevalence, % (95% credible interval)** | **Positive samples, method 2** | **Positive samples, % (95% CI), method 2^b^ (Wuhan & BA.2 antibodies)** | **Estimated seroprevalence, % (95% credible interval), method 2** | **Vaccinated with at least one dose of COVID-19 vaccine, (%). Data from reference no. 11** |
|  | | | | | | | |  |
| **0-11** | 366 (19.1%) | 297 | 81.1 (76.8 - 85.0) | 83.3 (79.0 - 87.5) | 339 | 92.6 (89.4 - 95.1) | 97.7 (92.6 - 100.0) | 2^c^ |
| **≥12** | 1,548 (80.9%) | 1,479 | 95.5 (94.4 - 96.5) | 98.2 (96.4 - 99.8) | 1,513 | 97.7 (96.9 - 98.4) | 99.5 (98.2 - 100.0) | 88.9 |
|  | | | | | | | |  |

^a^Method 1: seropositivity was based on having antibodies against both spike and receptor binding domain (RBD) from the Wuhan variant of SARS-CoV-2. ^b^Method 2: seropositivity was based on either having antibodies against both RBD and spike from the Wuhan variant of SARS-CoV-2 (as in Method 1) or having antibodies against Omicron BA.2 RBD. ^c^5-11 years only.

**Supplementary Table 3. Seroprevalence estimates by county of residence, 2022**

|  | | | | | | | |  |
| --- | --- | --- | --- | --- | --- | --- | --- | --- |
| **County** | **Number of samples tested (%)** | **Positive samples, method 1^a^** | **Positive samples, % (95% CI), method 1 (Wuhan antibodies)** | **Estimated seroprevalence, % (95% credible interval), method 1** | **Positive samples, method 2^b^** | **Positive samples, % (95% CI) method 1 (Wuhan & BA.2 antibodies)** | **Estimated seroprevalence, % (95% credible interval), method 2** | **Vaccinated with at least one dose of COVID-19 vaccine, (%). Data from reference no.16** |
|  | | | | | | | |  |
| **Oslo** | 118 (6.2%) | 107 | 90.7 (83.9 - 95.3) | 92.7 (86.5 - 97.8) | 115 | 97.5 (92.7 - 99.5) | 98.9 (96.1 - 100.0) | 90 |
| **Rogaland** | 127 (6.6%) | 120 | 94.5 (89.0 - 97.8) | 96.4 (91.3 - 99.8) | 125 | 98.4 (94.4 - 99.8) | 99.3 (97.4 - 100.0) | 90 |
| **Møre og Romsdal** | 139 (7.3%) | 134 | 96.4 (91.8 - 98.8) | 97.8 (94.0 - 99.9) | 136 | 97.8 (93.8 - 99.6) | 99.5 (97.9 - 100.0) | 92 |
| **Nordland** | 125 (6.5%) | 116 | 92.8 (86.8 - 96.7) | 94.8 (89.1 - 99.2) | 121 | 96.8 (92.0 - 99.1) | 98.9 (96.1 - 100.0) | 92 |
| **Viken** | 390 (20.4%) | 362 | 92.8 (89.8 - 95.2) | 95.4 (92.2 - 98.6) | 374 | 95.9 (93.4 - 97.6) | 98.9 (96.3 - 100.0) | 91 |
| **Innlandet** | 113 (5.9%) | 104 | 92.0 (85.4 - 96.3) | 94.0 (87.7 - 98.9) | 107 | 94.7 (88.8 - 98.0) | 98.5 (95.0 - 100.0) | 92 |
| **Vestfold og Telemark** | 141 (7.4%) | 126 | 89.4 (83.1 - 93.9) | 91.4 (85.2 - 96.6) | 137 | 97.2 (92.9 - 99.2) | 98.8 (95.7 - 100.0) | 91 |
| **Agder** | 101 (5.3%) | 94 | 93.1 (86.2 - 97.2) | 94.9 (88.6 - 99.3) | 98 | 97.0 (91.6 - 99.4) | 99.0 (96.2 - 100.0) | 91 |
| **Vestland** | 239 (12.5%) | 225 | 94.1 (90.4 - 96.8) | 96.5 (93.0 - 99.4) | 233 | 97.5 (94.6 - 99.1) | 99.2 (97.3 - 100.0) | 91 |
| **Trøndelag** | 209 (10.9%) | 185 | 88.5 (83.4 - 92.5) | 90.8 (85.8 - 95.3) | 195 | 93.3 (89.0 - 96.3) | 98.0 (93.3 - 100.0) | 93 |
| **Troms og Finnmark** | 197 (10.3%) | 191 | 97.0 (93.5 - 98.9) | 98.4 (95.5 - 99.9) | 196 | 99.5 (97.2 - 100.0) | 99.7 (98.8 - 100.0) | 91 |
| **Missing** | 15 (0.8%) | 12 | 80.0 (51.9 - 95.7) | n.a. | 15 | 100.0 (78.2 - 100.0) | n.a. | n.a. |
|  | | | | | | | |  |

^a^Method 1: seropositivity was based on having antibodies against both spike and receptor binding domain (RBD) from the Wuhan variant of SARS-CoV-2. ^b^Method 2: seropositivity was based on either having antibodies against both RBD and spike from the Wuhan variant of SARS-CoV-2 (as in Method 1) or having antibodies against Omicron BA.2 RBD. n.a.: not applicable. Seroprevalence was not estimated if a subgroup had less than 30 samples. ^c^Percent individuals ≥16 years.

**Supplementary Table 4: Median neutralization titer of residual sera subgroups**

| **Virus variant** | **Subgroup**^a^ | | |
| --- | --- | --- | --- |
|  | **W^+^BA2^-^ (n=20)** | **W^+^BA2^+^ (n=20)** | **W^-^BA2^+^ (n=20)** |
| **B.1** | 142 | 1824 | 44 |
| **BA.2** | 8.8 | 790 | 509 |
| **BQ.1.1** | 5.0 | 43 | 89 |
| **XBB.1.5** | 5.0 | 34 | 49 |

^a^Subgroups of residual sera from the late summer of 2022 selected based on their ability to inhibit ACE2-RBD_W, ACE2-RBD_BA2 interaction or both. W: RBD of Wuhan type, BA2: RBD from Omicron BA.2 (BA2). + indicates high inhibition, - indicates no/low inhibition

**Supplementary Table 5. Seroprevalence estimates for cohort participants**

|  | | | | | | | |
| --- | --- | --- | --- | --- | --- | --- | --- |
| **Age (years)** | **Number of samples tested (%)** | **Positive samples, method 1^a^ (Wuhan antibodies)** | **Positive samples, % (95% CI), method 1** | **Estimated seroprevalence, % (95% credible interval)** | **Positive samples, method 2^b^** | **Positive samples, % (95% CI), method 2 (Wuhan & BA.2 antibodies)** | **Estimated seroprevalence, % (95% credible interval), method 2** |
|  | | | | | | | |
| **All** | 243 (100%) | 210 | 86.4 (81,5-90.5) | 88.8 (83.8 - 93.4) | 240 | 98.8 (96.4-99.7) | 99.5 (98.0 - 100) |
| **≤12** | 90 (37.0%) | 59 | 65.6 (54.8 - 75.3) | 67.1 (57.0 - 76.5) | 87 (96.7) | 96.7 (90.6- 99.3) | 98.6 (94.2 - 100) |
| **>12** | 153 (63.0%) | 151 | 98.7 (95.4 - 99.8) | 99.0 (96.6 - 100) | 153 | 100.0 (97.6 - 100) | 99.9 (99.4 - 100) |
|  | | | | | | | |

^a^Method 1: seropositivity was based on having antibodies against both spike and receptor binding domain (RBD) from the Wuhan variant of SARS-CoV-2. ^b^Method 2: seropositivity was based on either having antibodies against both RBD and spike from the Wuhan variant of SARS-CoV-2 (as in Method 1) or having antibodies against RBD from Omicron BA.2.
